# Development and Evaluation of a Smart Contract–Enabled Blockchain System for Home Care Service Innovation: Mixed Methods Study

**DOI:** 10.2196/15472

**Published:** 2020-07-28

**Authors:** Shuchih Ernest Chang, YiChian Chen, MingFang Lu, Hueimin Louis Luo

**Affiliations:** 1 Graduate Institute of Technology Management National Chung Hsing University Taichung Taiwan

**Keywords:** home care service, trust, innovation, blockchain, smart contract, automation

## Abstract

**Background:**

In the home care industry, the assignment and tracking of care services are controlled by care centers that are centralized in nature and prone to inefficient information transmission. A lack of trust among the involved parties, information opaqueness, and large manual manipulation result in lower process efficiency.

**Objective:**

This study aimed to explore and demonstrate the application of blockchain and smart contract technologies to innovate/renovate home care services for harvesting the desired blockchain benefits of process transparency, traceability, and interoperability.

**Methods:**

An object-oriented analysis/design combined with a unified modeling language tool was used to construct the architecture of the proposed home care service system. System feasibility was evaluated via an implementation test, and a questionnaire survey was performed to collect opinions from home care service respondents knowledgeable about blockchain and smart contracts.

**Results:**

According to the comparative analysis results, the proposed design outperformed the existing system in terms of traceability, system efficiency, and process automation. Moreover, for the questionnaire survey, the quantitative analysis results showed that the proposed blockchain-based system had significantly (*P*<.001) higher mean scores (when compared with the existing system) in terms of important factors, including timeliness, workflow efficiency, automatic notifications, insurance functionality, and auditable traceability. In summary, blockchain-based home care service participants will be able to enjoy improved efficiency, better transparency, and higher levels of process automation.

**Conclusions:**

Blockchain and smart contracts can provide valuable benefits to the home care service industry via distributed data management and process automation. The proposed system enhances user experiences by mitigating human intervention and improving service interoperability, transparency/traceability, and real-time response to home care service events. Efforts in exploring and integrating blockchain-based home care services with emerging technologies, such as the internet of things and artificial intelligence, are expected to provide further benefits and therefore are subject to future research.

## Introduction

### Background

Nowadays, human resource management has become a critical issue in the home care industry owing to the advent of an aging society and the growing proportion of double-pay families. The inability to adequately care for elderly people and those with disabilities has created a growing demand for home caregivers. While care centers act as intermediaries in matching home care cases with suitable home caregivers, existing procedures require substantial manual processing (eg, individual case matching, deployment/working status tracking, and employee insurance processing). Consequently, it has become difficult for the existing system to build trust relationships among service providers (care centers), care providers (caregivers), and caretakers [1].

Additionally, majority of the current service platforms store relevant data in their respective local databases, which subsequently leads to information asymmetry, opaqueness, and tampering. These issues have led to a lack of trust among individual systems and have mitigated the level of automation. Moreover, requests must be made to care service providers to retrieve the latest service or insurance status information. Therefore, it is difficult to obtain up-to-date tracking information regarding process flows. The lack of traceability, transparency, and trust among participants increases user concerns (eg, fairness of deployment, insurance status checks, decision-making regarding services provided, and classification/evaluation of care service providers and home caregivers). A practical and radical solution is not only beneficial but also promising for developing a sound home care industry ecosystem [2].

### Blockchain and Smart Contracts

Blockchain, as a distributed ledger technology, may solve the aforementioned issues along with its affiliated technology smart contracts. A blockchain can be viewed as a consecutive chain of blocks wherein transaction records are stored. By virtue of its data storage and consensus algorithm, data authenticity and verification are maintained by participating nodes and the distributed network [3,4]. This allows shared duplicates against malicious tampering and results in a trustless operational environment without centralized trusted third parties [5]. Blockchain may provide benefits through innovation, but there may be uncertainty due to technical limitations [6]. However, blockchain allows transparent and auditable transaction records with chronical time stamps, and participants are able to trace related transactions and information flow [7]. Typically, blockchain can help facilitate medical data management [8-11] and drug tracking against potential counterfeit [12].

A smart contract is a computer protocol that can be encoded to digitally facilitate, verify, or enforce the terms or agreement of a contract. Smart contracts have the following characteristics: (1) self-verifying, ability to prove to an arbitrator that a contract has been performed; (2) self-enforcing, enforcement of contractual clauses when predetermined rules/conditions are met; and (3) tamper proof (because of deployment on the blockchain network). Smart contracts may enforce preset rules, implying the potential exemption of trusted third parties. Smart contracts may transform contract terms into programmable logic, with automatic execution if the preset conditions are met [13]. Smart contracts may therefore replace a part of human operation and facilitate more automatic process executions [14]. Ethereum, a popular blockchain platform, supports smart contract execution for various applications by using a Turing-complete language (Solidity) [15,16]. Smart contracts can be automatically executed by programmable codes without manual operation, which, in the long term, can avert human errors and allow workflow automation to achieve higher levels of efficiency [17]. In addition, security is attained because of the tamper-proof characteristics, whereby a more trustful operating system can be established. Certain misconceptions regarding smart contract adoption have been reported [18], including methods regarding the storage/retrieval of data from the blockchain [10,19,20]; however, academic endeavors have not yet discussed how smart contracts can improve process automation in the home care industry.

To facilitate process automation leveraging smart contracts, an event-driven mechanism has been introduced. The event-driven mechanism is a way for the computer system and its affiliated components to manage/handle the required process information flow. It allows applications to communicate with, detect, and react to events. Events can be viewed as a kind of state change. For example, in a home care service scenario, caregivers accept the case, and the state of service matching may change from “undetermined” to “matched.” The occurrence of an event could be further transmitted to applications in the architecture for process managing purposes. A typical pattern is the widely known publish/subscribe or emitter/receiver mechanism. Events are emitted by publishers and received by subscribers who had registered earlier. In this sense, home care event messages are transmitted via functional calls of smart contracts to enable asynchronous communication among system components and computer nodes, and thus, they facilitate procedural manipulation of the overall home care service.

Owing to technical limitations, only data with permanent attributes are considered worthy to be recorded in blockchain because unnecessary storage may increase resource consumption and transaction costs [21]. Generally, existing data required for blockchain transactions may be stored in offline databases, and the interoperability among different systems becomes a critical issue for information exchange [22,23]. The data required by a blockchain system may be retrieved from offline databases by using gateways [24]. For security and privacy concerns, corporations may integrate offline databases with their own developed gateways [25]. In practical implementation, it may be more reasonable to develop self-owned gateways to bridge data access across on- and off-chain systems.

### Existing Service Process and Blockchain Roles

Traditional paper-based processes and manual scheduling of service assignments have been heavily adopted in the home care industry. Typical service procedures include service matching, schedule planning, and optimal assignment/dispatch in accordance with critical indicators (available service vacancy, caregiver specialty, and case conditions). Traditional care centers are responsible for manual processing, while specialized matching systems assist by providing potential matching results. However, the incumbent system may still suffer from poor efficiency and heavy processing for queries and responses.

**Figure 1:**
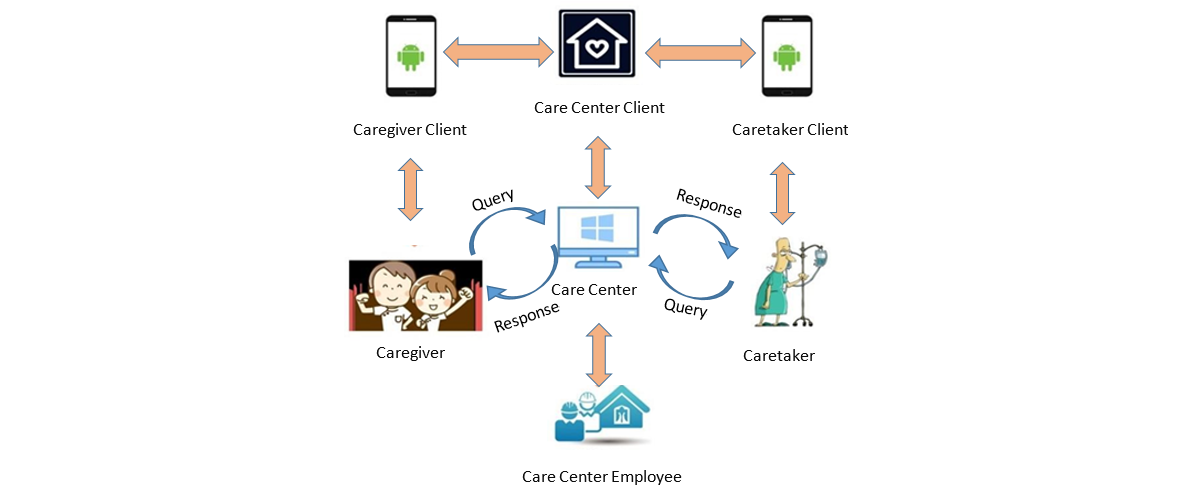
Overview of the current matching system.

The major participants of home care services include caretakers, caregivers, and care centers (Figure 1). Each participant has individual siloed databases for data storage. A care center has to confirm and communicate with other units to acquire the latest home care status in that timely updated information is not available from the individual systems of the other two participants. In a short-term employment scenario, a care center may apply for insurance for its caregivers to cope with emergencies or unexpected circumstances. Insurance companies have been reluctant to provide relevant short-term products owing to the complexity of the application/cancellation process and costs related to manual processing. While accidental events may be inevitable during service delivery, caregivers’ employment and demands from care recipients may be influenced by the lack of insurance products in the home care industry.

**Figure 2:**
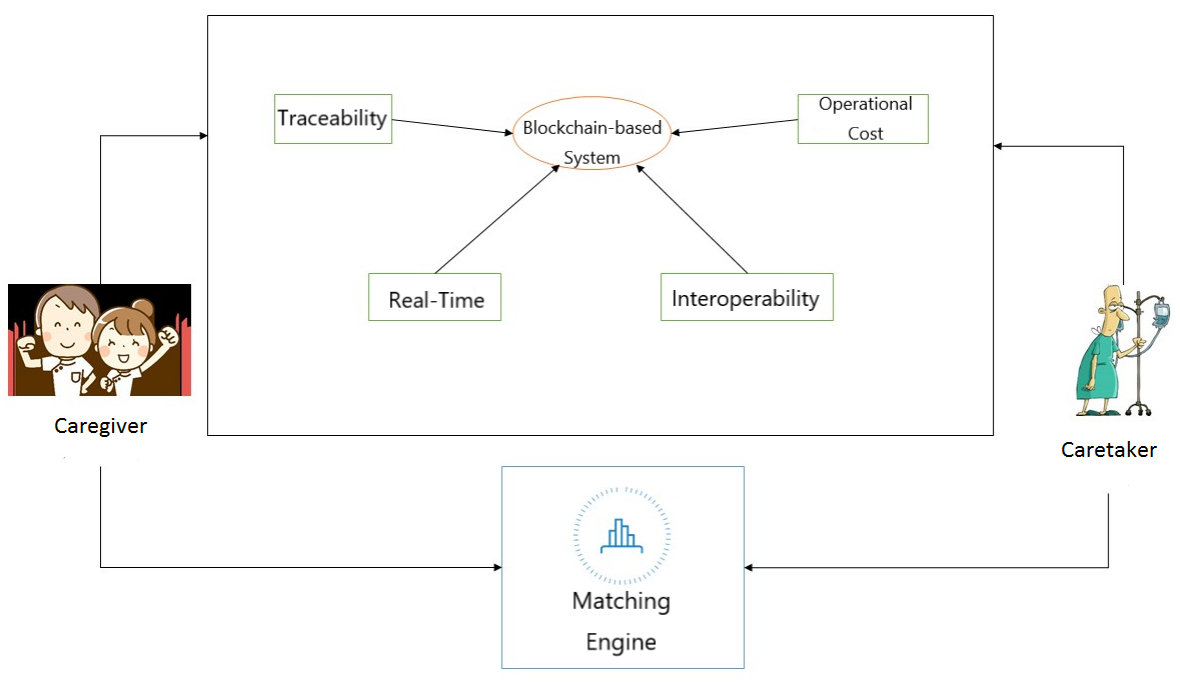
The role of blockchain in improving the home care service system.

Owing to blockchain data storage and consensus algorithms, home care service participants may benefit from a common shared ledger and enjoy process automation facilitated by smart contracts. Figure 2 illustrates the roles that blockchain and smart contracts could play in improving the current issues faced by the home care industry in terms of traceability, timeliness, interoperability, and cost. The proposed design may reduce the degree of human intervention, thereby increasing the integrity and performance of the system during operation. Relevant smart contracts are designed to connect the entire system’s process flow, enabling the automation of service processes. Moreover, parties may perceive a higher level of freedom to request status information, which in turn enhances transparency and traceability during the service process. Additionally, the event mechanism of smart contracts can instantaneously disseminate information to all parties and enable response to important events.

### Study Objective

A potential blockchain-based solution (ie, a blockchain and smart contract–enabled home care service system) is proposed to resolve the aforementioned issues affecting the home care industry. We not only developed a step-by-step system design protocol but also evaluated the core functions and the proposed automated processes to test the system’s feasibility.

## Methods

### Overview of the Blockchain and Smart Contract–Enabled Home Care System

**Figure 3:**
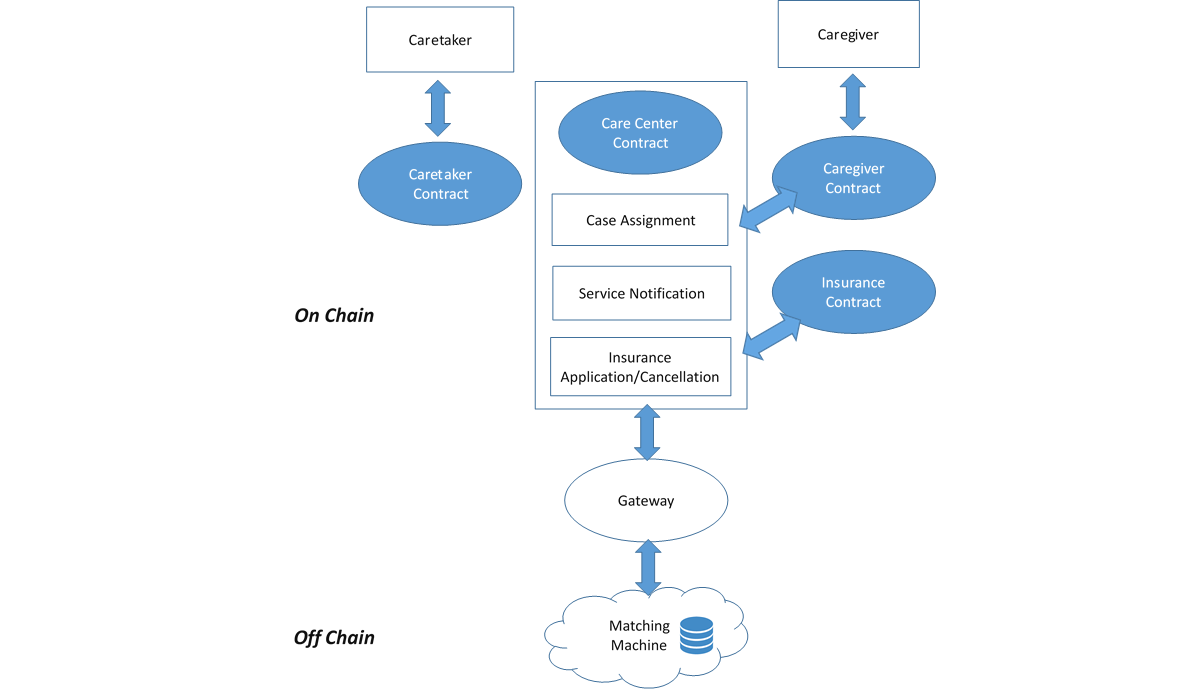
Overview of the blockchain and smart contract–enabled home care system.

To address the major issues in delivering home care service in terms of case assignment, information notification, and insurance procedures, our research team consisting of the authors and two research assistants (programmers) worked part-time and developed a blockchain-based home care service system. The study took place from October 2017 to January 2019. From a process re-engineering perspective, we combined the unique features of blockchain and smart contracts in the design of an integrated system to solve major pain points using an existing home care system. Figure 3 shows the proposed framework of the blockchain-based home care service system. The system’s operation begins by receiving matching cases and enabling the automatic deployment of caregivers. A caretaker receives automatic notifications after a caregiver’s acceptance. The caretaker may either decide to start the home care service or not, and the system may automatically enroll/cancel the insurance for the caregiver. A potential solution is designed and explored to achieve greater process efficiency and timeliness in terms of caregiver matching, task assignment, service notification, and insurance-related processes. The proposed design allows improved transparency/visibility on process status transitions by introducing event-driven smart contracts on a blockchain-based platform. Additionally, preset criteria for insurance claims are designed to activate the claims process once triggering conditions are met.

For the above-described purposes, this study focused on the interactions among care centers, caregivers, caretakers, and insurers, and thereby designed the following four kinds of smart contracts: care center contract, caregiver contract, caretaker contract, and insurance contract. The proposed system integrates with the existing matching engine, which provides optimal matches, and retrieves off-chain matching results via a gateway for further use in the care center contract. This contract deals with task assignment, service notifications to caregivers/takers, and insurance-related processes.

### Process Flows and Business Model of the Proposed System

**Figure 4:**
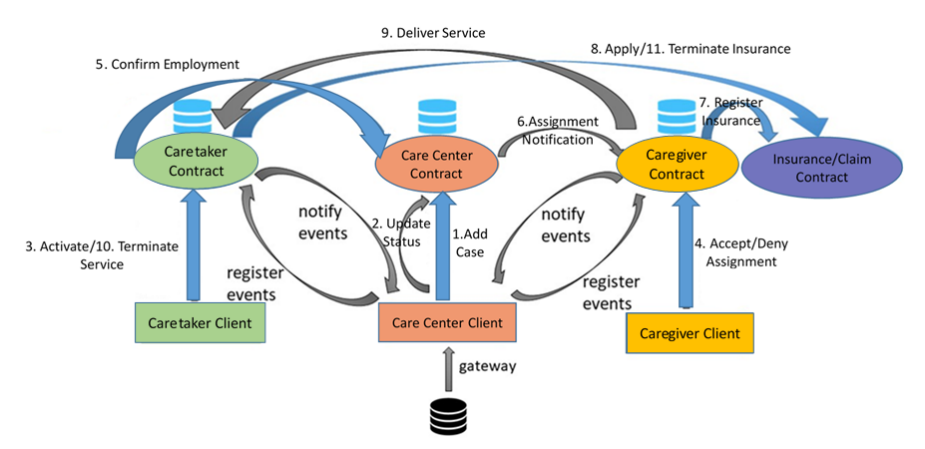
Framework of the proposed blockchain-based home care system.

Figure 4 presents the framework for the proposed system to elucidate the design of smart contract interactions from a project management perspective. Home care service participants may track the system’s status in a timely manner and be informed of data relevant for decision-making via event-driven notifications. Smart contracts were further utilized to design an insurance claims procedure that may simplify the complex manual review processes and enable claims procedures to be automatically executed.

#### System Process Flow

Initially, an external matching engine uploads service-matching results and related information to the care center contract through a gateway via a standard information interface provided by the system. Thereafter, care center employees dispatch task assignments to the caregiver contract. Automatic assignment notifications are then forwarded to the caregiver for his/her final decision. The caregiver contract receives the caregiver’s response and then delivers a confirmation message (regarding accept/reject results) to the care center contract. Once an assignment is confirmed, the care center contract executes the notification function previously coded in the caretaker contract and informs the caretaker of related service information. The caretaker may decide to accept/reject the service. If the caregiver accepts, he/she makes a request to activate the home care service. Meanwhile, the care center contract creates an insurance application for the caregiver. When the home care service is completed, the caretaker can ask the care center contract to terminate the home care service.

This system utilizes smart contracts to connect holistic service processes. The event-driven mechanism allows decision information to be transmitted to the relevant decision makers. Once decisions are made, functional linkage would be redirected to the original service processes. Despite the decision points, all service processes in this system are executed by corresponding smart contracts without manual intervention.

#### New Business Model

The proposed system can serve as a public platform for improved service status tracking and as a potential solution for caregiver insurance issues.

#### Blockchain-Based Platform

The existing home care service system adopts a centralized model for operational management. This study presents a new type of business model for participating stakeholders. In the current home care system, an individual caregiver/taker makes requests to either public sector or social service units for case matching and passively waits for vague matching results. Thereafter, authorized care centers take charge of care service processing till the end of the service. This existing model suffers from a lack of transparency and poor efficiency owing to its centralized operation, making it difficult to establish a fair and impartial evaluation mechanism.

The proposed system seeks to provide a solution that simultaneously favors both the caregiver/taker and care centers. First, caretakers may not be equivalent in their economic capabilities. Existing social service providers, religious/charity parties, and public sector organizations have been undertaking related projects to meet the increasing demand from stakeholders. The proposed system allows for the inclusion of home care services in these projects by providing a new operational model for developing a fair and open matching platform. Apart from professional caregivers, volunteer caregivers can also be incorporated to extend the home care service capability to support policy. In these projects, it is technically feasible to add new categories of caregivers in project implementation without incurring too much overload in terms of computing algorithms and system design. This operational model can be designed by the integration of on- and off-chain databases. While the matching engine enables massive data processing by using complex algorithms, computational processing may be assigned to the offline system to generate the final matching results for upload to the on-chain system.

The proposed system can be treated as a service execution engine that is capable of (1) tracking the status of caregivers’ assignment and execution process, (2) automatically notifying caretakers about service commencement and dominant rights regarding service initiation/termination, and (3) introducing newly developed insurance applications for improving caregivers’ personal protection.

#### New Insurance Products Based on the Blockchain System

**Figure 5:**
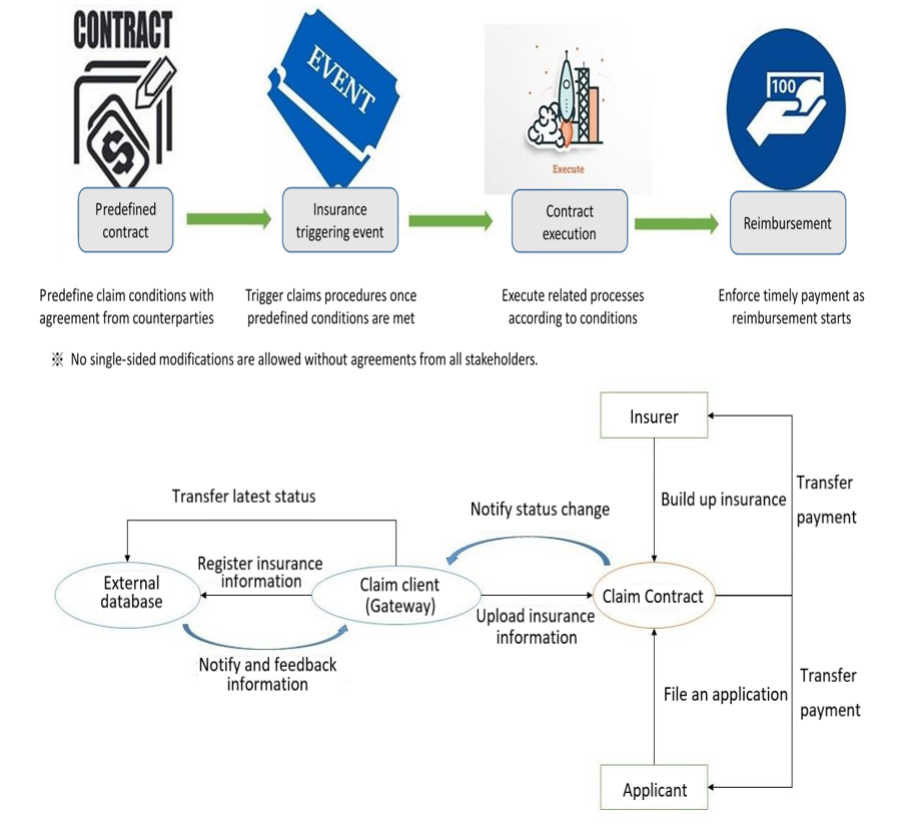
Framework of the smart contract–enabled insurance claims procedure.

Traditional insurance claims procedures may take weeks or even months for reimbursement owing to the massive volume of manual processing involved. Increased administrative costs may lead to a higher premium for applicants. A smart contract–enabled insurance process transforms enforcement terms and rights and obligations into programmable codes. Once an insurance event has occurred, such as a traffic accident, corresponding information can be uploaded to the claims contract, which in turn makes a comparison with preset claims conditions. If these conditions are met, claims proof-related information is recorded on the blockchain and automatic claims procedures are activated. The reimbursement is automatically executed without any manual intervention (Figure 5). In this regard, smart contract–enabled insurance may not only reduce considerable administrative costs/time but also provide transparent disclosure on claimed items and criteria. The overall claims procedures are open to all stakeholders and are enforced according to contract terms. Such an insurance application may develop better trust among stakeholders, thereby ensuring their rights and obligations.

In practice, the insurance company automatically files the corresponding insurance policy whenever a home care case is confirmed and established by the care center contract according to a comprehensive evaluation of caregiver/caretaker background and other risk considerations during service delivery. The subsequent process is enabled by an insurance smart contract accordingly, and claims for the caregiver are conducted whenever insurance conditions are met with status changes/updates.

This study explored a new insurance product wherein no particular insurer is necessarily required to act as an insurance carrier since the main design rationale is to set methods of developing insurance policies and filing insurance applications as payable functions. The use case of the claims contract is generated by insurers, which enables policy reserves to be deposited in the contract. When applicants file insurance applications, the policy reserves are deposited in the claims contract. Once a claim’s conditions are met, the proposed system automatically executes the settlement of loss reserves from the total policy reserves according to the claims ratio between applicants and insurers. This allows for the exemption of manual review/auditing procedures with automatic claims enforcement in place against intended noncompliance.

### Object-Oriented Methodology and Unified Modeling Language Analysis

**Figure 6:**
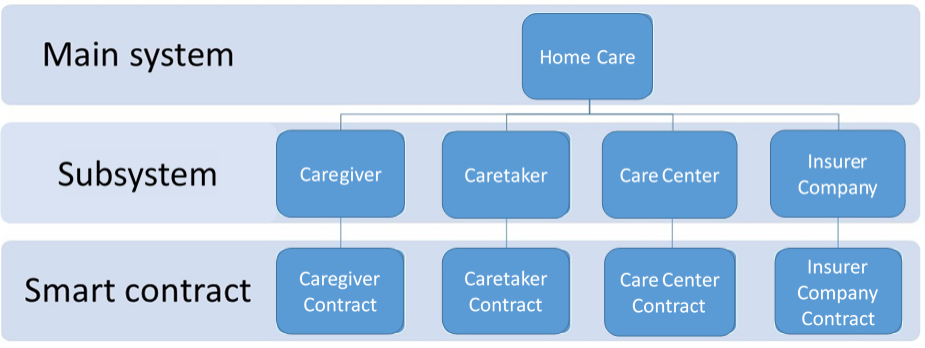
Architecture of the home care service system.

This study uses object-oriented application/design methodologies for the system analysis and design [26,27]. Unified modeling language diagrams were utilized for system modeling [28]. In unified modeling language, use case diagrams are used to represent system functions and the interactions between the actors and these functions. In a static configuration, class diagrams are used to describe the data structures of smart contracts and the methods by which users could access their services. In a dynamic configuration, sequence diagrams are used to depict how actors interact with smart contracts to fulfil compulsory functions and to specify the operational processes of the system. More detailed steps of system design are provided in Multimedia Appendix 1. The home care system comprises four subsystems with affiliated smart contracts (ie, caregiver, caretaker, care center, and insurer contracts) (Figure 6). The proposed framework enables short-term home care service assignment, automatic process state notifications to stakeholders, and insurance application/cancellation by incorporating the insurers’ contracts.

To better present the feasibility of the proposed system, we conducted an implementation test to demonstrate the functionality of each system component and operational process. More detailed test guidelines, strategies, and procedures are provided in Multimedia Appendix 2.

### Implementation and Validation

#### Implementation Platform

This study’s implementation aimed to validate the feasibility of the smart contract–enabled home care service system. Since front-end platforms require further study, the development of the blockchain system, including the validation of interoperability and status transitions among smart contracts, has become more important. The Chrome browser (Google) and Remix software with a JavaScript VM environment were required for system validation. We utilized Solidity for smart contract programming and Solidity compiler 0.4.18 for the compilation of Solidity contracts that were deployed in the blockchain system for further testing [29].

#### Implementation Procedures

The following assumptions were made before implementation to simplify the procedures of feasibility validation: (1) For each caretaker, only one care service is accepted by a caregiver on a daily basis; (2) A caregiver only provides service to one caretaker; and (3) A care center provides only one insurance application per caretaker and caregiver.

### Comparative Analysis of the Existing and Proposed Systems

A step-by-step comparison of care service workflow was performed to point out differences between the existing and proposed systems. In particular, the examination in terms of critical relevant dimensions (including transparency, traceability, level of automation, counterfeit/fraud proofing, and management of insurance/welfare) may help shed light on the competitive advantages to be availed of when adopting a blockchain-based system. The blockchain and smart contract features are major criteria for giving qualitative measures on individual steps. Through step-by-step workflow comparisons, valuable information of system comparisons was extracted to provide overall insights from a qualitative perspective.

### Questionnaire Survey

In this study, we also administered a questionnaire survey to investigate the feasibility of a blockchain-based system by conducting comparisons with the existing system. The selected respondents were participants in a final term exhibition of the Digi+ Talent Accelerator & Jumpstart Program, administered by the Industrial Development Bureau, Minister of Economics Affairs, Taiwan. This incubator program is a 4-year program, and the first-year program was completed during the period from July 1 to December 31, 2017. The exhibition gathered Fintech scholars, engineers, and practitioners in blockchain-related fields from academia, industry, and public sectors in Taiwan. Participants were selected and filtered during enrollment to make sure that they were qualified with matching and had appropriate knowledge and experience, particularly related to blockchain and home care services. The questionnaire was validated by an expert panel invited from the selected respondents, while data were collected in December 2017 and analyzed in early 2018. The surveyed aspects of the questionnaire were divided into the following five major constructs: the care center, caregiver, caretaker, insurer, and public sector. The four items of traceability, efficiency, automation level, and management were considered critical for measuring the feasibility of the proposed system. Purposive sampling was adopted for scholars, experts, practitioners, and graduate students, and a total of 50 home care service respondents were collected in a 7-day period. The selected respondents were considered suitable for the questionnaire survey as they were familiar with home care services and were knowledgeable about blockchain technology and smart contracts.

## Results

### Results From the Implementation Test

**Figure 7:**
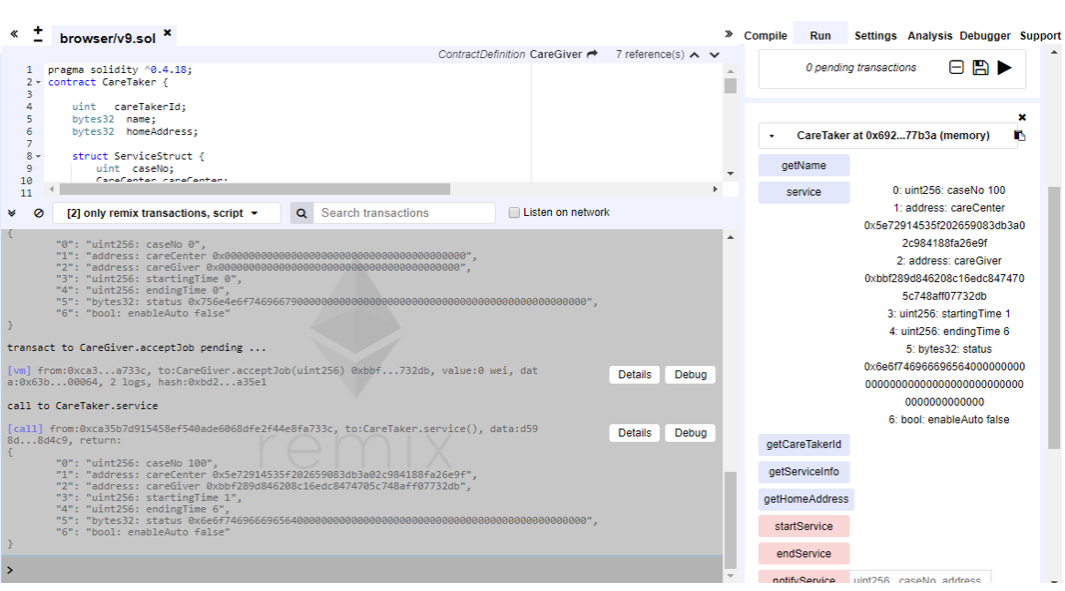
Illustration of the caretaker contract status after task assignment.

**Figure 8:**
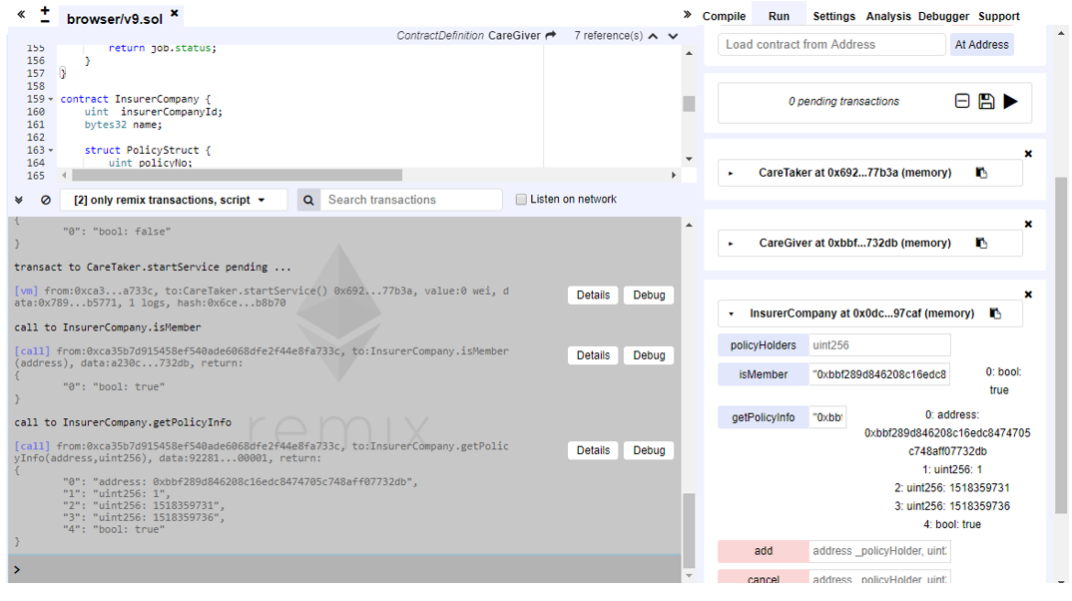
Illustration of the insurance application status after service activation.

For every use case scenario, we developed class codes to implement needed service flow as described in the previous section and also detailed in Multimedia Appendix 1. Actually, we conducted all necessary functionality tests to ensure that the implemented service flow functions smoothly as expected by design. The service flow is home care case establishment, task assignment, service notification, service activation, automatic insurance application, service termination, and insurance cancellation. While testing all service flow sequences, we extracted related testing screenshots, which are shown in this paper and in Multimedia Appendix 2, to demonstrate that interactions among smart contracts correctly match the designed service flow scenarios. In this paper, we only present two test result–related screenshots (Figures 7 and 8) to demonstrate the real interactions among smart contracts with service status changes. More comprehensive test result–related screenshots together with their matching service flow scenarios are presented in Multimedia Appendix 2.

Figure 7 illustrates the caretaker contract status after task assignment. The care center automatically notifies the caretaker of related service information upon receiving caregiver acceptance. Again, by using the service function, related service information is available in the bottom corner of the right-hand side column. For example, case number 100 is reported with care center and caregiver address details. Figure 8 presents the notification and status of the insurance policy after service activation. The system is designed in a manner such that the care center may automatically file an insurance application for the caregiver once the care service is activated. Users could inquire about the insurance-related status by using the getPolicyInfo function to get insurance policy details.

### Illustrations From Comparative Analysis

To have a better understanding of how blockchain and smart contracts enable improving performance against the existing system, we conducted a step-by-step comparison of the care service workflow for reference (Multimedia Appendix 3). In addition, we compared the proposed system with the existing system in terms of five major constructs (Table 1). The major differences were reduced administrative costs, increased automation, and timely tracking of system status.

**Table 1:** Comparison of constructs between the existing system and the proposed system.

Construct	Existing system	Blockchain-based system
Transparency	1. Highly centralized governance structure in single home care authority 2. Lack of trust due to unavailability of monitoring 3. Discrepancies in auditing/evaluation	1. Smart contract-enabled management and review of aggregated information and evaluation indices 2. Information transparency with public-access authority 3. Improved trustworthiness of the overall system
Counterfeit	1. Manual interference during operations 2. Difficulties in waiving artificial alterations and possibility of tampering	1. Replacement of manual operations by smart contracts 2. Mitigation of alterations and tampering of previous recorded data due to permitted access control
Traceability	1. Job vacancies by carer queries 2. Manual notification to the case 3. Update latency on the latest status	Automatic notification by an event-driven mechanism
Level of automation	Manual processing on case assignment, notifications, and insurance-related procedures	Smart contract–enabled process automation
Insurance and welfare	1. Complexity regarding the application/cancellation of existing products 2. Lack of insurance coverage in the home care industry	1. Newly developed insurance product to facilitate the review process in terms of claims proportion 2. Automatic claiming process and imbursement enabled by preset terms or agreement 3. Large reductions in administrative costs and better insurance offers for applicants

### Findings From the Questionnaire Survey

Since the existing and proposed systems are not completely independent of each other, this study utilized a dependent *t* test to analyze the mean differences. Given that the sample was normally distributed and possessed homogeneity of variance, this study conducted the analysis using a dependent *t* test. Table 2 reports the means and SDs for the existing and proposed blockchain systems in terms of the five major constructs. Further data analysis showed that significant differences existed (*P*<.001) and each function in the proposed blockchain system was superior to that in the existing system. The proposed blockchain system was therefore validated for better performance and excellence than the traditional system. More detailed information about the content of the questionnaire is provided in Multimedia Appendix 4.

This study analyzed the potential reasons for the most significant items in terms of their *t* values. For care centers, the respondents generally considered that the proposed system may outperform the existing system by providing better traceability of caregiver assignments and service status. For caregivers, the automatic notifications from the blockchain system provide them with automatic service status updates and notifications along with the subsequent activation of claims procedures. For caretakers, respondents think that improved efficiency of insurance claims procedures can be achieved since the system enables automatic insurance application/cancellation. For insurers, the interactive processes in the smart contract design allow companies to automatically execute short-term insurance application/cancellation and facilitate claims procedures. Finally, the public sector may benefit from blockchain’s immutable transaction records for easy tracking, case matching/assignment, and related insurance information.

**Table 2:** Comparison of construct scores between the existing system and the proposed blockchain-based system.

Item (function)					Existing system score^a^, mean (SD)		Blockchain system score^a^, mean (SD)		*t* value^c^
**Care center (beneficial to…)**									
	Traceability^b^				3.28 (0.991)		4.20 (0.639)		5.619
		Efficiency^b^				3.28 (1.070)		4.10 (0.763)	4.499
		Automation^b^				3.14 (1.088)		4.14 (0.857)	4.763
		Management^b^				3.18 (1.024)		4.08 (0.724)	5.161
**Caregiver (beneficial to…)**									
			Traceability^b^			3.24 (1.041)		4.28 (0.730)	6.340
			Efficiency^b^			3.24 (1.061)		4.14 (0.783)	5.306
			Automation^b^			3.10 (1.035)		4.26 (0.853)	7.029
**Caretaker (beneficial to…)**									
			Traceability^b^			3.12 (1.100)		4.12 (0.849)	5.052
			Efficiency^b^			3.00 (0.990)		3.96 (0.903)	6.354
**Insurer (beneficial to…)**									
			Traceability^b^			3.18 (1.063)		4.10 (0.678)	5.039
			Efficiency^b^			3.10 (1.055)		4.18 (0.691)	6.404
			Automation^b^			2.96 (1.009)		4.22 (0.648)	7.584
			Management^b^			3.16 (0.976)		4.22 (0.790)	5.986
**Public sector (beneficial to…)**									
				Traceability^b^	3.34 (0.939)		4.30 (0.647)		6.354
				Efficiency^b^	3.26 (1.065)		4.32 (0.621)		6.066

^a^Score ranging from 1 (strongly disagree) to 5 (extremely agree).

^b^*P*<.001.

^c^Degree of freedom df=49.

## Discussion

### Principal Findings

The proposed system contributes to the understanding of blockchain-based application in the home care service industry. The aforementioned system design, implementation, and testing showed that the system achieved the expected requirements in terms of delivering the needed home care service functionalities and harvesting the desired blockchain benefits. In the real world, when a critical change in service status occurs, related events are emitted to notify registered clients. These functional demands are actually achieved while the design of nonsynchronization may cope with functional circumstances in a real system. The structural design, development, implementation, and feasibility testing of the proposed blockchain-based system reflect its potential for home care service applications. The step-by-step design protocol also provides a reference for future academic research. However, for the change from prototype to real implementation in an actual home care scenario, major challenges and implementation issues need to be addressed and discussed to shed light on orientations in a future study.

### Practical Implementation Issues

Generally, the major challenges to blockchain implementation in the real world on a large scale going forward can be classified into technical, organizational, and environmental aspects [30], and we need to address such technical, organizational, and environmental issues to make our blockchain-based home care service actually work in real life. System efficiency is an extremely important technical challenge to address because the system design will not be practical unless the efficiency of our blockchain-based system is acceptable [31]. In terms of efficiency improvement, researchers suggested exploring various technological configurations on blockchain platforms, consensus protocols, data exchange mechanisms between on-chain and off-chain systems, block sizes, etc [31]. Cultural shift and mindset are also critical factors when organizations attempt to embrace blockchain applications. From paper-based procedures to digitization processes, high resistance from transformation needs to be either mitigated by employee training or ameliorated by more incentives and perceived benefits. In addition, the unwillingness for data disclosure among distributed parties may hinder large scale information sharing, thus affecting the overall performance of blockchain-enabled systems. For example, care providers may be reluctant to share data with insurers owing to the lack of concrete incentives and legacy system users may have security/privacy concerns with regard to adopting new technology. Finally, government policies or regulatory support for emerging blockchain technologies could be influential. The implementation processes for health care administrations could be affected by such environmental factors. Without proven credibility from leading pilot projects, large-scale adoption may not be viable and promising for various applicable industries to harvest desired blockchain benefits.

Specifically, to accelerate settlement and claims procedures for our blockchain-based home care service, the following two practical issues must be addressed:

- Claims or settlement conditions attested by a trusted third party: For example, as information of a traffic accident or personal safety may be recorded in the documents of a police agency, open data inquiry may be required via deregulation or rule adaption.
- Establishment of a gateway: A functional gateway, such as Oracle, is required to transfer external open data into the claims contract for on-chain use.

An alternative gateway design to achieve data exchange is feasible by using an observer pattern with the event-driven mechanism of smart contracts. The method can be configured as follows. The client of a claims contract registers the requested data category in the open data server. Automatic notifications to the contract client are executed when the requested data are available. The contract client may forward data to the claims contract. Similarly, the contract client may inversely transfer status changes via emitting events, which carry status values/parameters before or after an incurred event, to the client and then to external systems. Common tokens (ie, a kind of cryptocurrency, such as Ether in the Ethereum platform) could be used as payment tools among contract counterparties; however, this requires a sound exchange mechanism between flat currency and tokens. If tokens are sensitive to exchange rate fluctuations, it may be difficult to build up a stable and widely adopted industrial ecosystem. Thus, the issuance of a specific token for the home care industry may be required for function as a payment instrument, thereby solving the issue that existing cryptocurrency is currently unavailable for commercial transactions.

### Blockchain and Smart Contract–Enabled Applications in the Home Care Industry

We integrated the blockchain-based home care system with the existing home care service-matching engine. The matching between caretakers and caregivers was completed by the existing matching engine while matching results were implemented and utilized by the proposed blockchain system. Focused issues and functionalities of the integrated system are addressed and illustrated in Figure 2. This study identified major issues in the existing system, including traceability, timeliness, interoperability, and cost, and later, it investigated how these issues would be mitigated by innovated/renovated blockchain-based home care services.

#### Traceability

A lack of an adequate workforce may result from labor conditions, social image, and individuals’ expectations. Less than 30% of trained caregivers choose to stay in the home care industry, and the lack of an audit system with openness and trust is a major home care issue [32]. Consequently, the ineffective management of caregivers’ promotion mechanism and care center classification results in the potential loss of the home care workforce and poor trustworthiness of care centers. Therefore, the emphasis should be on recording and maintaining related data for establishing a better auditing/evaluation system. These data should be open access and be characterized by trust and immutability. The blockchain-based system in this study may provide better system trustworthiness compared with that of the existing system.

#### Timeliness

Home care assignments are dispatched to caregivers by manual notifications while the care recipients are informed of assignments later. Assignment procedures involve multiple participants with delays in information transmission and disputes regarding assignment fairness. This study utilized Solidity, a programmable language provided by the blockchain platform, along with an event-driven mechanism to formulate an automatic notification function [16] to reduce user queries and pending durations induced by the existing process. Better timeliness was achieved on service assignment and case notifications in the new system. Moreover, at the beginning of the home care service, smart contracts may activate insurance application/cancellation to protect caregivers’ rights. The timely notifications and insurance-related process automation allow more fluent operation and monitoring of service processes.

#### Interoperability

Care centers have long relied on siloed databases to manage service information. With individual matching methods, the home care industry lacks uniform data exchange formats/standards, thereby increasing switching costs [23]. Additionally, a lack of standard operating procedures has resulted in the use of various auditing/evaluation indices, which causes discrepancies in auditing procedures. Based on the above concerns, the proposed system presents a standard interface for data communication and allows matching results to be uploaded from the existing matching engine. From the proposed standard procedure for service assignment and care service, the system can collect equivalent information for assessment indices and allow the unification of the performance evaluation of care centers and caregivers.

#### Cost

Heavy reliance on manual operation of case assignment and notifications, and the lack of standard operating procedures may increase administrative and management costs. This study used a blockchain-based system and smart contracts to integrate the overall service processes. In doing so, all detailed tasks in processes were automatically completed by smart contracts and a great reduction in operational cost was achieved by virtue of this distributed process automation scheme enabled by smart contracts.

### Recap of the Advantages of Adopting Blockchain-Based Home Care Services

The results of comparative analyses between the blockchain-based system and the existing system clearly shed light on the general influences and potential benefits of a blockchain-based home care service system. Apart from offering better transparency and traceability that a blockchain system can achieve in common use cases, the distributed working paradigm facilitates data exchange among siloed databases of care providers, thus enhancing interoperability and reducing overall cost. Nevertheless, the findings from the questionnaire survey also provide nascent evidence on system feasibility and potentials from the individual stakeholder’s perspective.

### Conclusion

#### System Feasibility Based on Validation Results

The proposed system has the potential to enable task assignment and status tracking, decision control by the caretaker/caregiver, service notifications to relevant participants, and automatic insurance application/cancellation for caregivers. In the proposed system, the required core functions were implemented using four smart contracts, and they facilitate process automation by connecting related contracts. Additionally, a clear distinction between responsibility and accountability was achieved since decision control with the required information is granted to the caregiver/taker through the event-driven mechanism of smart contracts. According to the screenshots excerpted from the Remix implementation, validation indicators set prior to implementation were met exactly, thus proving system feasibility. Moreover, research findings from the questionnaire survey implied improved performance and excellence with the proposed system when compared with the existing system. Therefore, a blockchain-based home care service system may have potential for future applications.

#### Future Prospects

Based on the validation of system feasibility, future studies should pay more attention to the improvement of system effectiveness and operational management. Technically, blockchain, as a distributed ledger system, has a revocable feature once data are added to the chain. Therefore, data structures and computing algorithms for the implemented smart contracts are required for scalability. From a managerial perspective, an open and impartial auditing mechanism should be created under evaluation of the care center and caretakers. The evaluated information must be stored on the blockchain with further classification of caregiver and wage standards to formulate a positive cycle. Caregivers may therefore have more economic incentive to provide better home care service. This evaluation mechanism can be made more reliable by virtue of blockchain’s open and tamper-proof characteristics. Evaluation information is open access to home care system participants such as caregivers, caretakers, and care centers. With better understanding of how service providers are assessed under specific standards, caregivers may have a better chance to accept or improve their services.

## Appendix

Multimedia Appendix 1 Detailed methods of system design.

Multimedia Appendix 2 System implementation.

Multimedia Appendix 3 Step-by-step workflow comparisons between the existing and proposed systems.

Multimedia Appendix 4 Home care service system questionnaire.
