# Supplementary material for: Development and Evaluation of a Smart Contract–Enabled Blockchain System for Home Care Service Innovation: Mixed Methods Study
Source: JMIR Med Inform. 2020 Jul 28;8(7):e15472. doi: 10.2196/15472 (PMC7420632; doi:10.2196/15472)
Supplement: Multimedia Appendix 1 [file medinform_v8i7e15472_app1.docx]

**Multimedia Appendix 1.** Detailed Methods of System Design.

**Overview of the System Structure and UML Analysis**

### Object-oriented Methodology and Unified Modeling Language Analysis

### *System Analysis and Design*

Use case diagrams were used to represent system functions and the interactions between the actors and these functions. In a static configuration, class diagrams are used to describe the data structures of smart contracts and the methods by which users could access their services. In a dynamic configuration, sequence diagrams are used to depict how actors interact with smart contracts to fulfill compulsory functions and to specify the operational processes of the system.

### *Analysis of System Functions/Processes and Use Case Development*

Figure 1 shows the use case diagram of the proposed homecare service system to illustrate the actors in and interactions among the four sub-systems. The actors include caregivers, caretakers, the care center, insurer company contracts, and the end-users of the sub-systems. An individual use case diagram is depicted to simulate system implementation, which includes the following processes: task assignment, assignment acceptance, service notifications, and insurance application/cancelation. Case matching is not included but is provided by matching results from the care center. End-users of sub-systems should deploy related smart contracts on the blockchain and utilize contract-provided functions to enable interactions among the use case actors.

After being deployed on the blockchain, smart contracts in the individual sub-systems execute the function create() to establish contracts in the system. Prior to task assignment, the end-user at the care center uses the function addCase() to transfer related matching results to the system and executes the function assign() for task assignment. The function assign() invokes the caregiver contract to execute the function receiveJob() to pass on information to the caregiver contract, which in turn sends event notifications to the caregiver. The caregiver may use the provided function acceptJob() to confirm if the assignment is accepted. Next, the caregiver contract executes the function assign() provided by the care center contract. The function notifyService() executed by the caretaker contract transmits service information to the caretaker contract via notifications and an event is emitted to inform the caretaker of the activated service. When the caregiver is about to provide service to the caretaker, the caretaker end-user may initiate the service by executing the function startService() provided by the caretaker contract. The caretaker contract may not only request that the care center contract activate the care service by calling the function enableCase() but also ask his/her insurer contract to complete an insurance application by executing the function add(). A caretaker end-user may invoke the function endService() to end the homecare service while also requesting the care center contract to terminate homecare service by executing the function disableCase(). The insurance cancelation procedure is initiated by the execution of the function cancel() by the insurer company contract.

**Figure 1.** Use case diagram of the homecare system


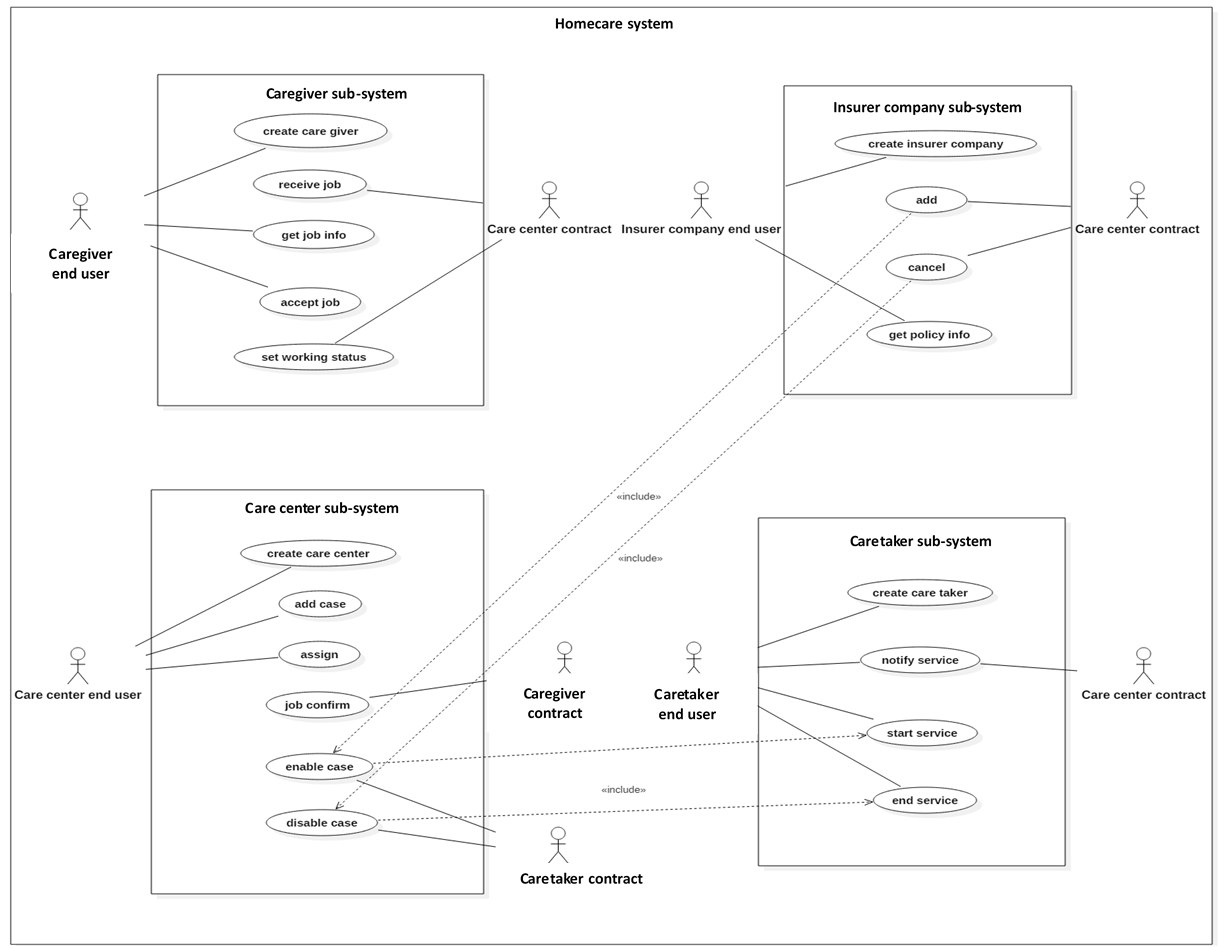


### *Static Architectural Design (in Terms of a Class Diagram)*

While use case diagrams describe the system’s basic functions and actors at the conceptual stage, the class diagram in the design phase depicts the component data structures and operational interface that it provides. The class diagram describes the relationships among objects, which include the inheritance, composition, and use relationships. The diagram is capable of expressing static structure. Generally, actors in a use case diagram may become class candidates of the class diagram. Similarly, the use cases in the use case diagram may become candidate methods in the class diagram. Figure 2 presents the system framework in the design stage via a class diagram.

**Figure 2.** Class diagram of the homecare system


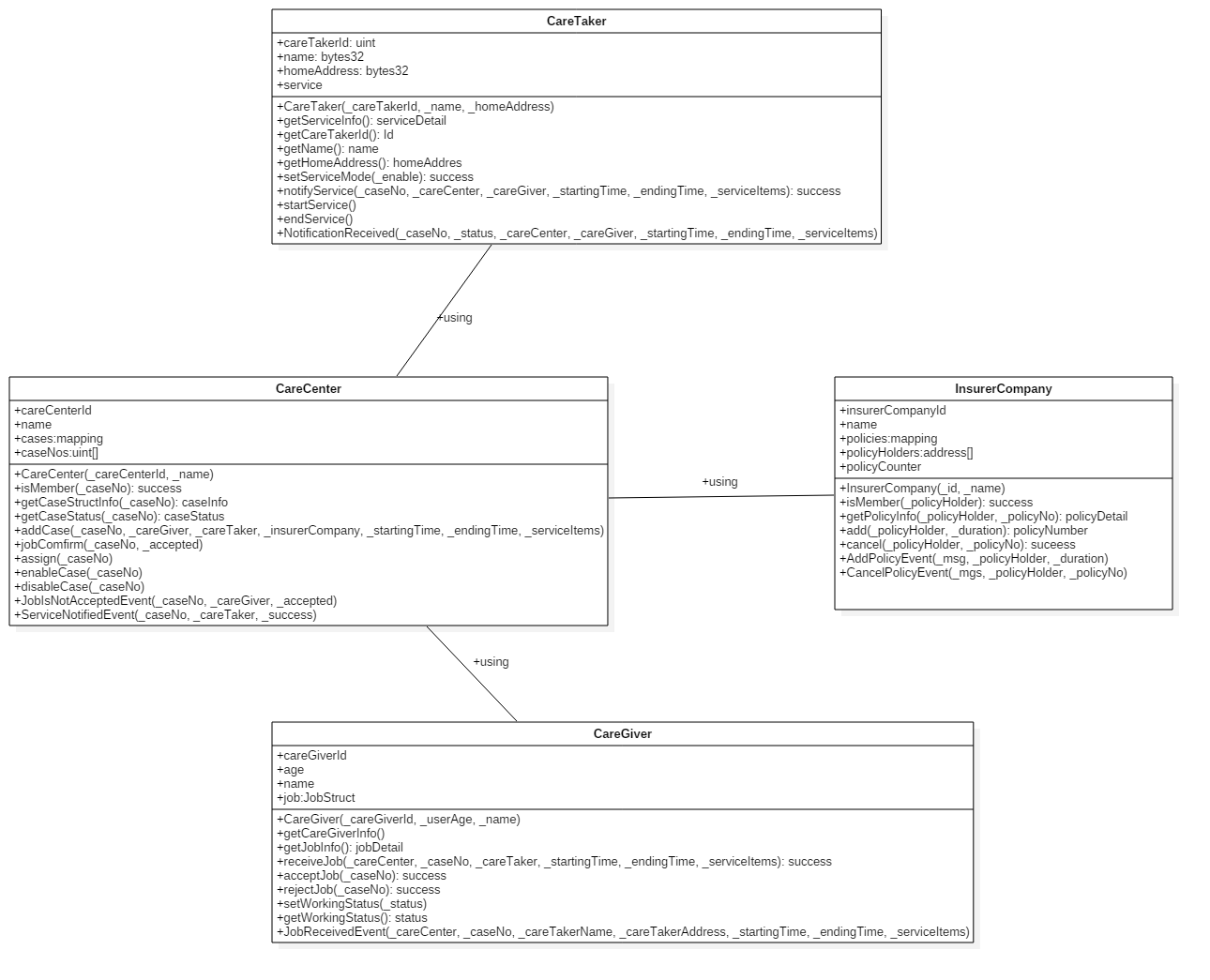


### *Dynamic Architectural Design (in Terms of a Sequence Diagram)*

Before implementation, it is necessary to determine the type of interactions among objects necessary for a specific function, along with the direction and sequence of information flow and the preliminary conditions that must to be satisfied. This data and information could be elaborated in sequence diagrams that represent specific system functions or a blueprint of the class methods. Using the care center sub-system as an example, Figure 3 illustrates how smart contracts and their end client users may interact with one another.

The care center end-user executes the createCareCenter() function to create a care center contract in the blockchain system and use the addCase() function to import off-chain matching data to the contract. The caregiver uses the jobConfirm() function to confirm the assignment and care center end-user uses the assign() function to dispatch tasks. The caretaker contract would initiate the service by using the enableCase() function provided by the care center contract, which in turn asks the insurer company to create an insurance application by executing the add() function. Additionally, the caretaker uses the disableCase() function to terminate the service case while the care center contract uses the cancel() function to ask the insurer company contract to execute the insurance cancelation procedure. Finally, the setWorkingStatus() function, provided by the caregiver contract, correspondingly changes the caregiver working status to offWork.

**Figure 3.** Sequence diagram of the care center sub-system


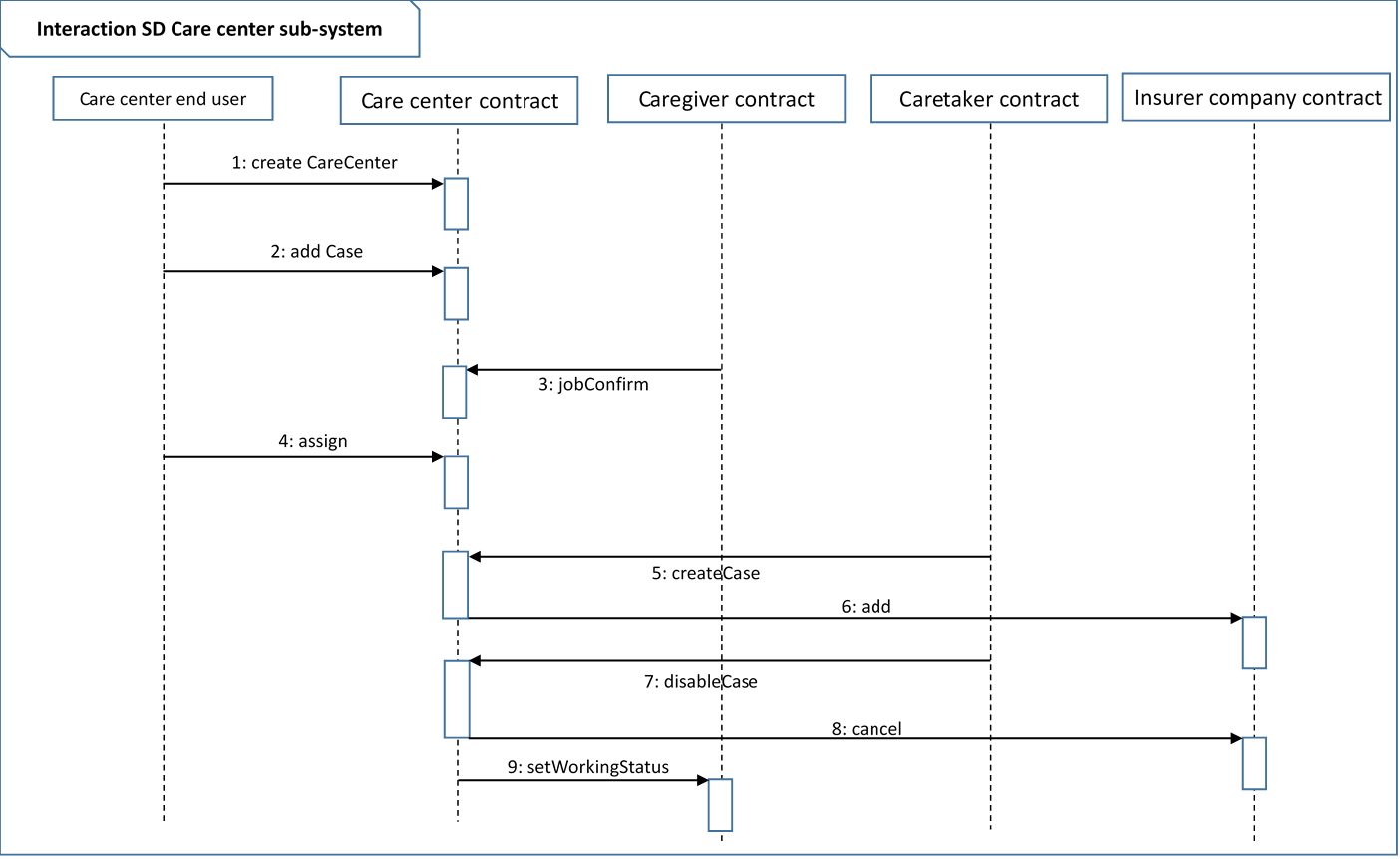


In this study, an insurance company follows a preset policy plan, which is confirmed by the care center contract. The proposed system enforces claim procedures when contractual conditions are met. For example, a caregiver accepts his/her service assignment and takes a ride to reach the location. The policy plan is packaged according to critical claim criteria offered by the insurance company when the service case is matched, such as service duration, transportation method, and potential risks generated due to care service. Once potential risks happened to the caregiver or caretaker, for example, an accidental traffic accident or medical/care malpractice, status update could be activated by either stakeholder to trigger the insurance claims procedures.
