# Supplementary material for: Development and Evaluation of a Smart Contract–Enabled Blockchain System for Home Care Service Innovation: Mixed Methods Study
Source: JMIR Med Inform. 2020 Jul 28;8(7):e15472. doi: 10.2196/15472 (PMC7420632; doi:10.2196/15472)

**Multimedia Appendix 2.** System Implementation.

### *Platform and Tool Selection Strategy*

The conception of the blockchain-based application’s design is to maintain data accessibility and trustworthiness without extra tools. A simplified system design may thus be achieved without loss of fairness. Two unique characteristics of blockchain technology are the sustainability of transaction records and immutability against tampering. These features allow transaction tracking and ensure the originality/integrity of transaction data. Additionally, by leveraging smart contracts, this study may improve the programmable capability of blockchain implementation and its diverse applications beyond a pure cryptocurrency platform. Clients utilize an application interface (API) provided by web3.js lib to manipulate smart contract functions via the JSON RPC protocol. The server uses Ethereum as an implementation platform as it supports a Turing-complete language, Solidity, which enables the coding of smart contracts.

### *Indicators of Validation and the Test Environment*

In a validation process, three objectives must be observed: (1) the real-time state of the smart contracts during system operation, (2) client notifications when smart contracts provide event and related information, (3) state changes in smart contracts when functions are executed. A final objective is that each contract function should be able to be manipulated by the client and follows system design procedures. Process execution should progress in the manner described in state diagrams to ensure operational accuracy.

In terms of cost, the test environment must provide an Ether account and unlock the designated account for actual transaction and validation testing. Moreover, direct compilation and deployment of smart contracts are required. The software, Remix IDE, may meet the above-described requirements and provides three parameter setting options: JavaScript VM, Injected Web3, and Web3 provider, which may be applied to virtual blockchain environments, non-local nodes environments (e.g., mobile phone platforms), and platform environments with local nodes, respectively. Remix can intercept all events emitted by its deployed smart contracts. Additionally, Remix IDE can act as a we3.js client to intercept any emitting event, thereby being able to observe actual interactions among contracts and clients. For the aforementioned reasons, this study used JavaScript VM in Remix to play as a client for the compilation, deployment, and execution of smart contracts. Remix may also provide validation if the execution results meet previously set objectives by examining contract states, major function states before/after execution, and events triggered by status changes.

Screenshots presenting critical workflow points and system functionalities are provided below:

(1) Create four use cases of smart contract

Figure 1 presents the creation of four use cases of smart contract. All web3.js clients deploy the four smart contracts on the blockchain while the required information is transmitted to contract constructor in terms of parameters. A contract address, serving as a compulsory parameter for inter-contract or contract-client interactions, is generated when contract is successfully deployed.

**Figure 1.** Illustration of creating four use cases of smart contract


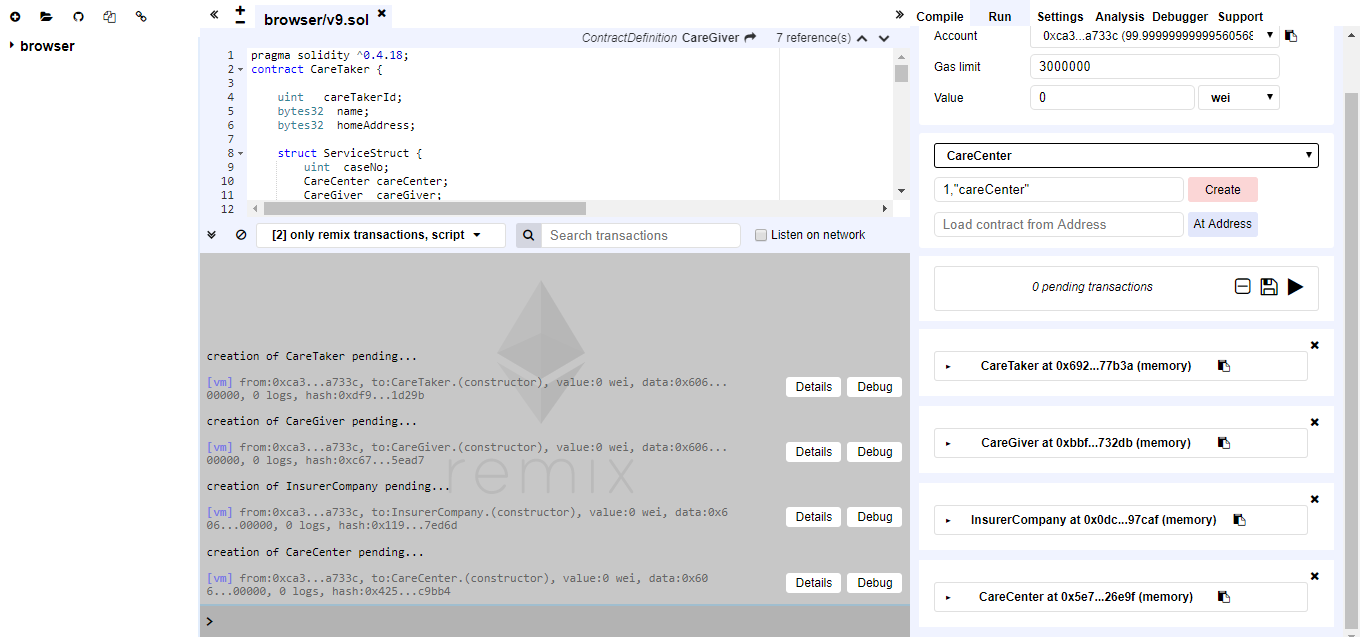


(2) Add case in the care center contract

Using the function addCase(), the care center may include matching information of new cases into the system and track its status during the service lifecycle (Figure 2).

**Figure 2**. Add care service case by care center contract


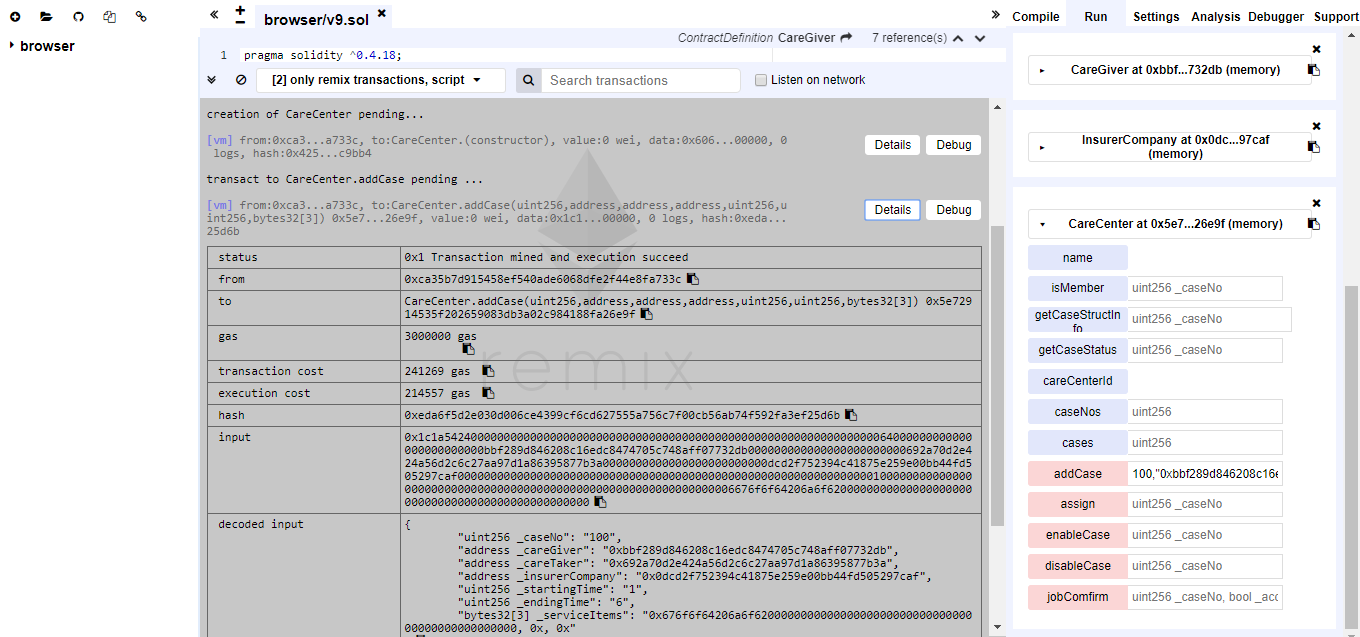


(3) Caregiver contract status prior to task assignment

Before using function assign( ) to dispatch service tasks, we may use function getJobInfo( ) provided by caregiver contract to inquire assignment status (Figure 3). The job column is left blank for stating the unassigned status.

**Figure 3.** Caregiver contract status prior to task assignment


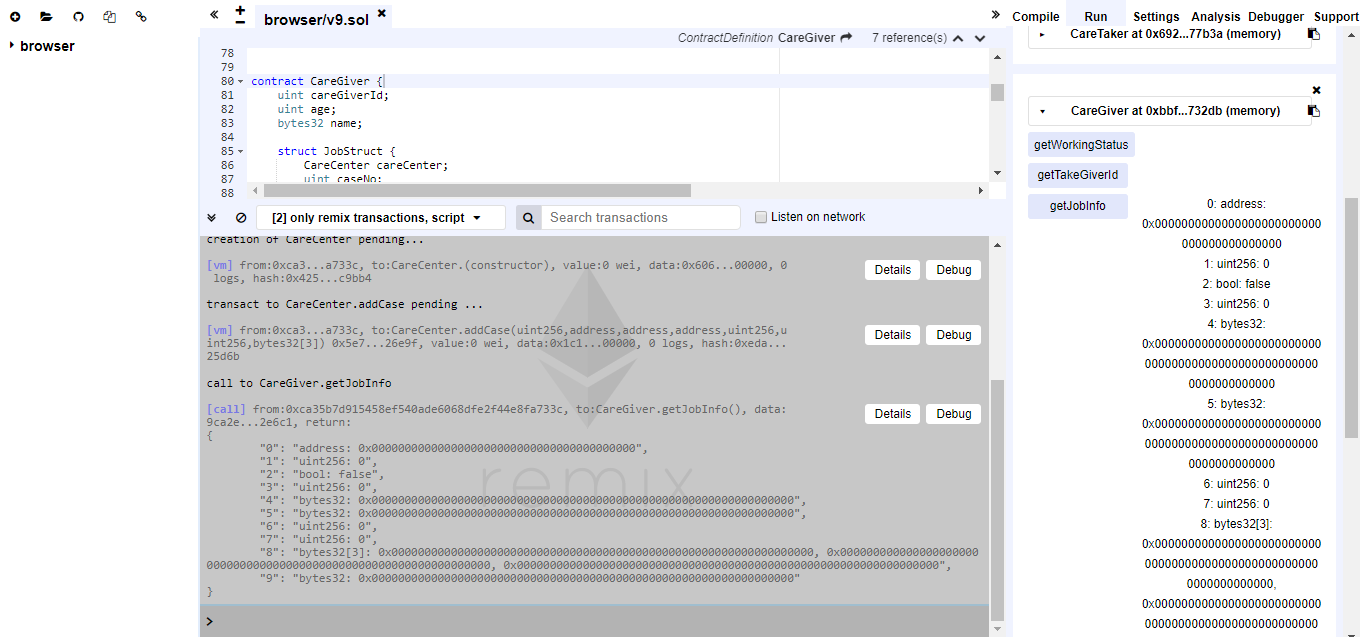


(4) Caregiver contract status after task assignment

When a task is assigned using the function assign(), a corresponding change of status is presented. The care center inputs the assignment information to the caregiver contract. Using the function getJobInfo(), we may examine the assignment list and related information (Figure 4).

**Figure 4.** Caregiver contract status after task assignment


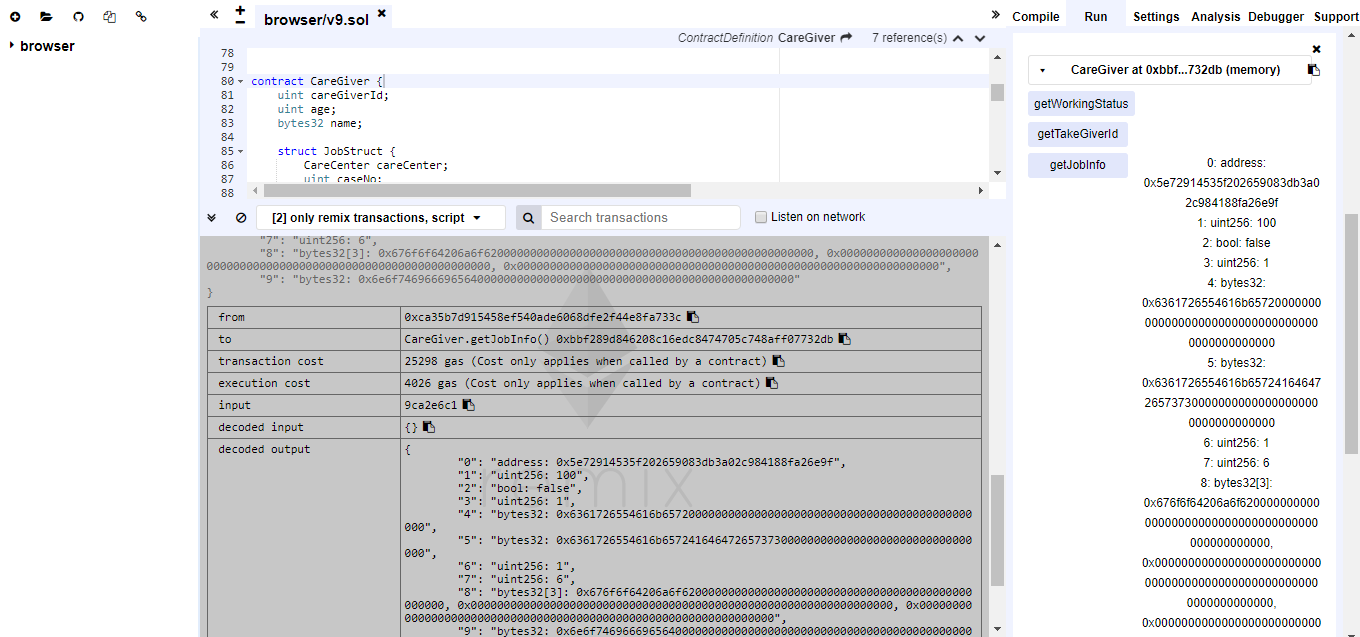


(5) Caretaker contract status before task assignment

No related information is presented without caregiver’s acceptance when the function service( ) from caretaker contract is executed (Figure 5). The proposed system takes the time gap (from assignment to acceptance) into consideration. System operation at the pending duration is non-synchronized (always a time delay) as there’s no immediate acceptance upon task assignment in a real practice.

**Figure 5.** Caretaker contract status before task assignment


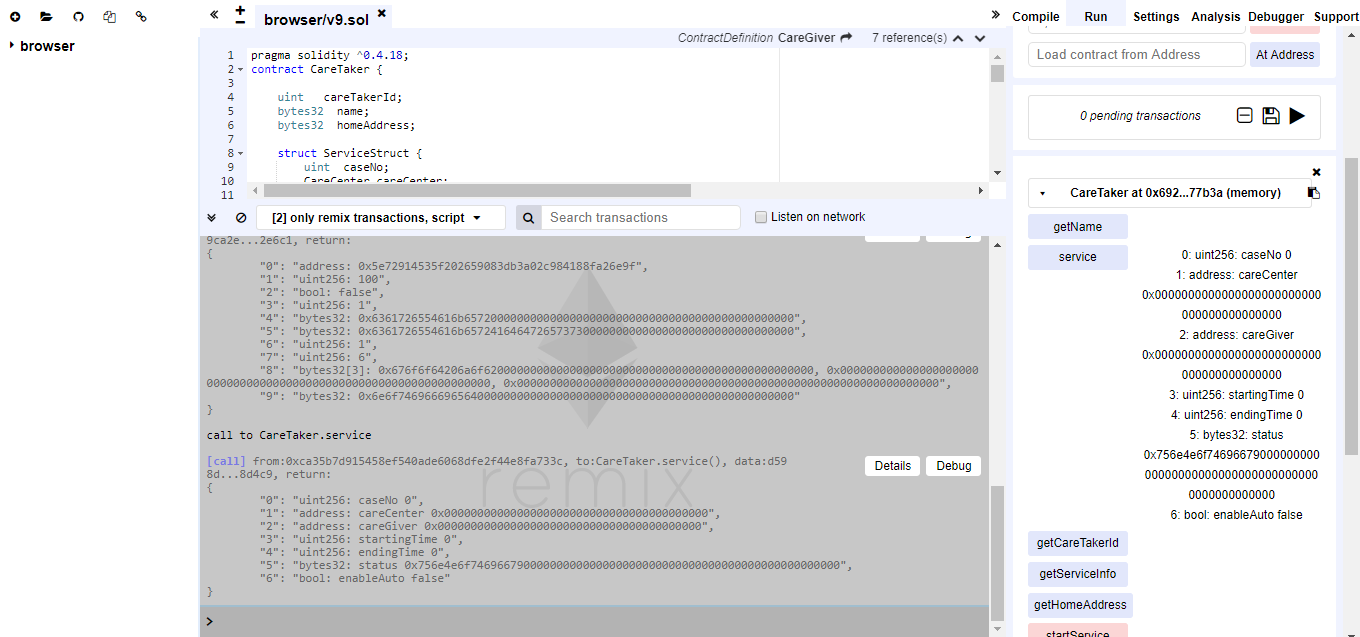


(6) Caretaker contract status after task assignment

Figure 6 illustrates the caretaker contract status after task assignment. The care center automatically notifies the caretaker of related service information upon receiving caregiver acceptance. Again, using the function service(), related service information is available in the bottom corner of the right-hand side column. For example, case number 100 is reported with address details of care center and caregiver.

**Figure 6 (Figure 7 in main text).** Illustration of Caretaker contract status after task assignment


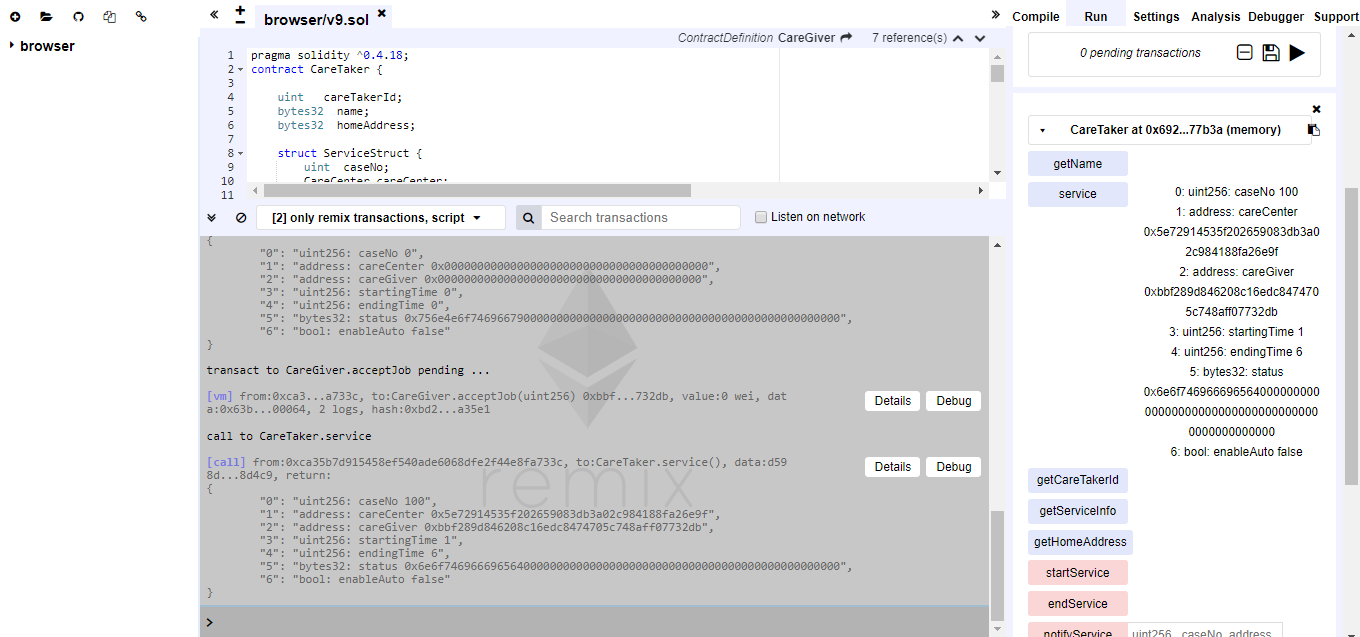


(7) Insurance status before service commencement

Without service activation by the caretaker, no insurance is applied by the care center and the caregiver is by no means registered as an insured member. Figure 7 shows the status of insurance application by using the function isMember( ). A response with Boolean “False” is reported accordingly.

**Figure 7.** Insurance status before service commencement


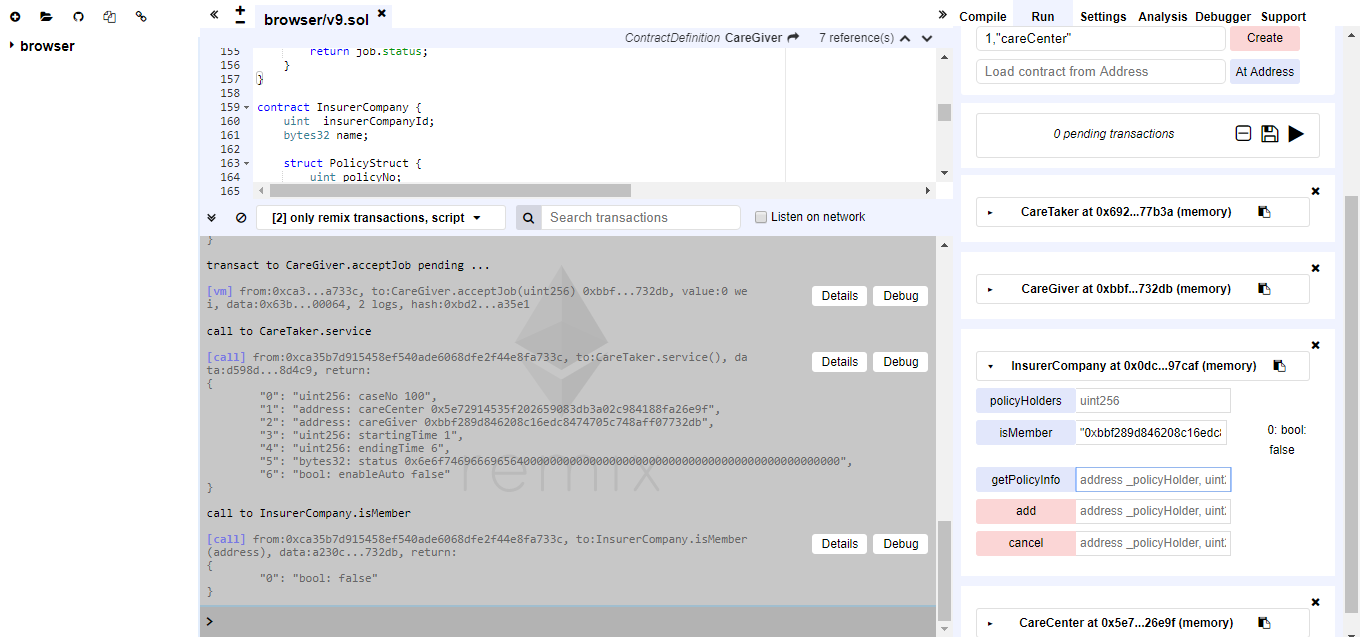


(8) Notification and status of insurance policy after service activation

The system is designed in a way such that the care center automatically files an insurance application for the caregiver once the care service is activated. The insurance sub-system would emit “AddPolicyEvent” (Figure 8). Figure 9 presents the notification and status of the insurance policy after service activation. Users could inquire into the insurance-related status by using the getPolicyInfo()function to get insurance policy details (reported Boolean “true” in system logs).

**Figure 8.** Notification of a successful insurance application after service activation


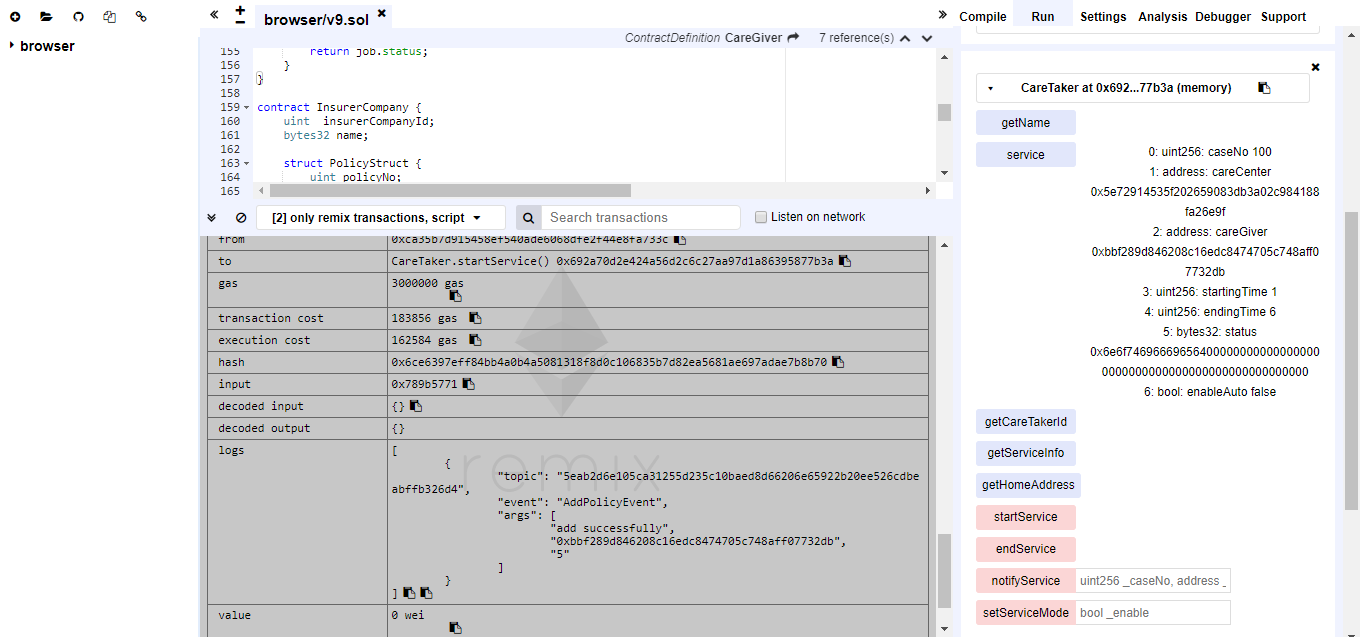


**Figure 9 (Figure 8 in main text).** Illustration of the status of the insurance application after service activation


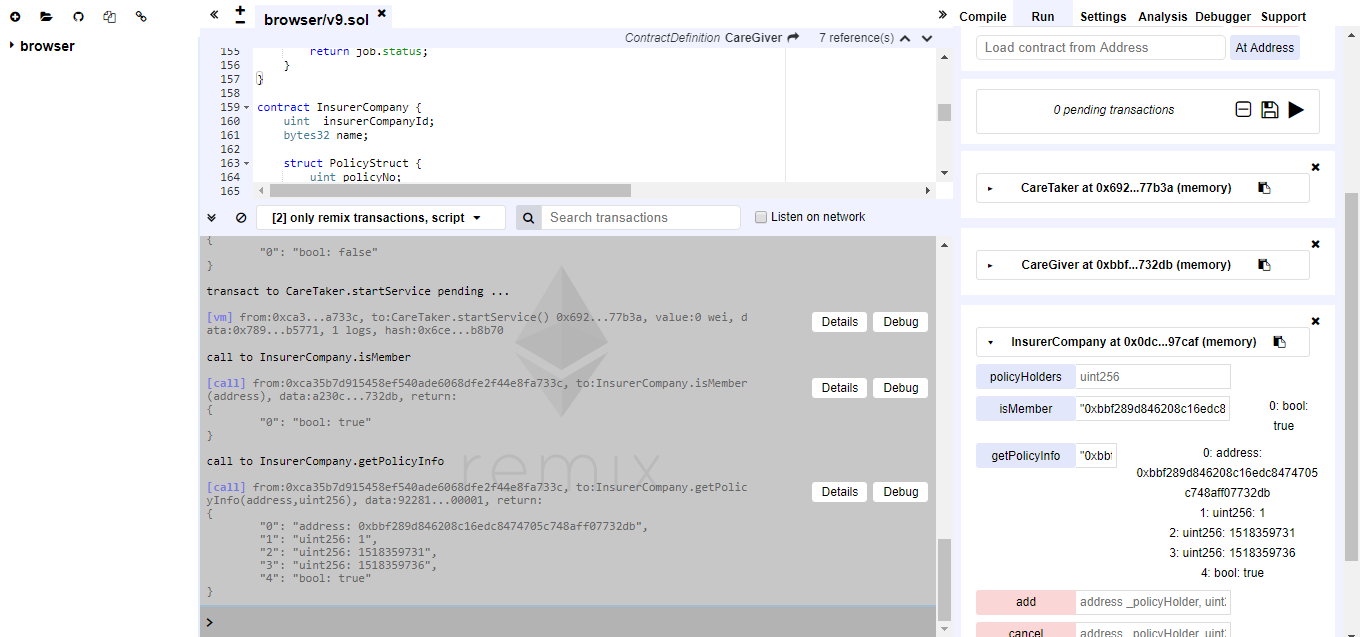


(9) Notification and status of insurance policy after service termination

When caretaker terminates the care service, care center would take over the procedure and make a request to insurance sub-system for executing insurance cancellation. Once insurance is cancelled, insurance system would in turn emit “CancelPolicyEvent” to notify caregiver (Figure 10). The insurance status could be checked subsequently, for example, reporting all 0s in related column information refers to the accomplishment of insurance cancellation (Figure 11).

**Figure 10.** Notification of a successful insurance cancellation after service termination


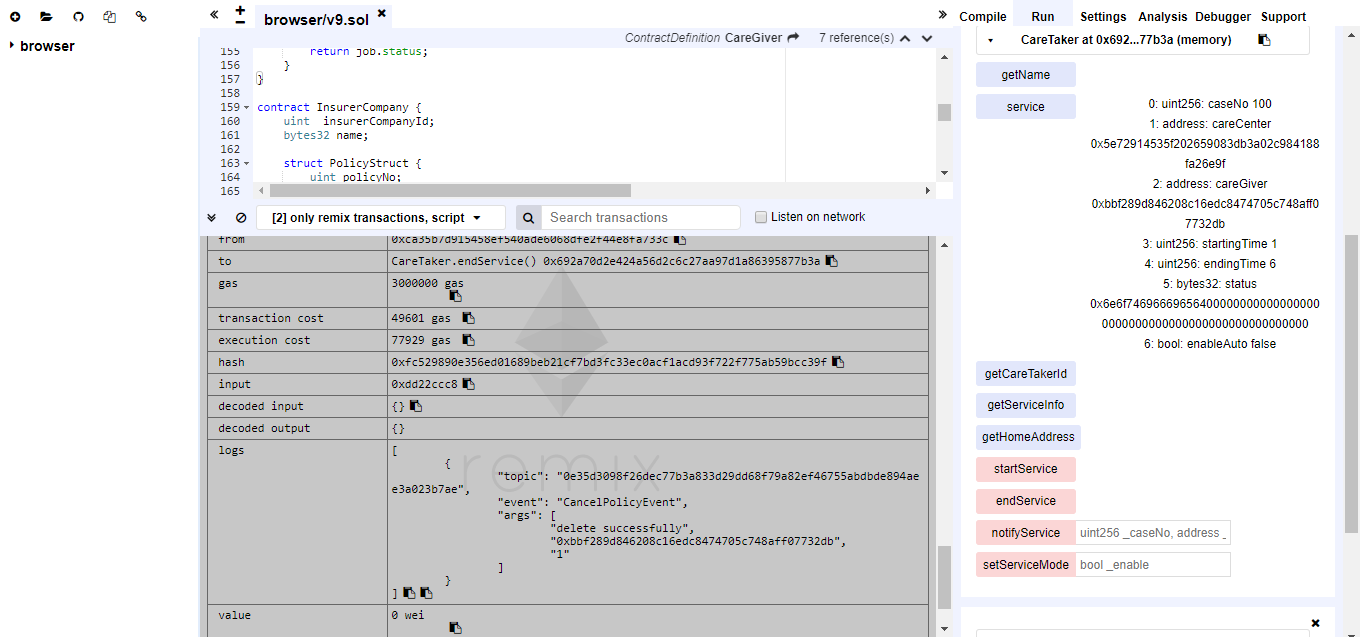


**Figure 11.** Status of insurance policy after service termination


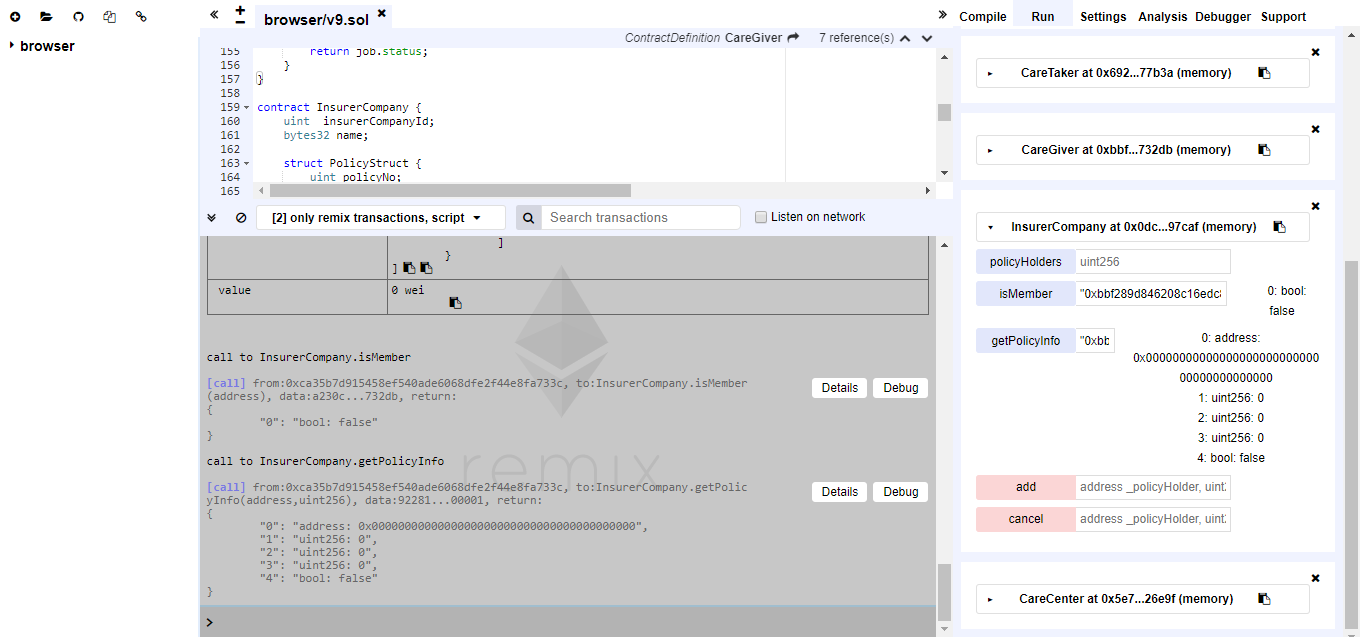


(10) Caregiver working status after case closed

Timely notification is sent to caregiver when care center terminates the care service. Status value is available by checking returns from function getWorkingStatus( ) (Figure 12 shows the returned value “0”).

**Figure 12.** Caregiver working status after case closed


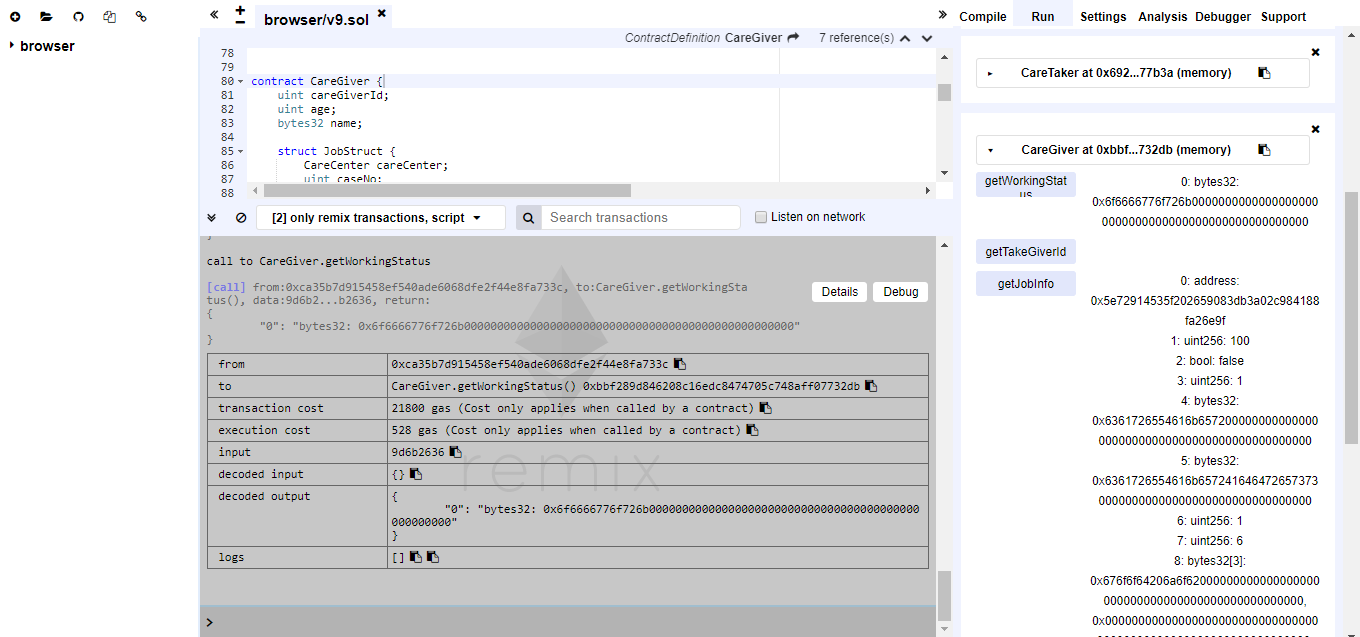

Supplement: Multimedia Appendix 2 [file medinform_v8i7e15472_app2.docx]
