# Supplementary material for: Development and Evaluation of a Smart Contract–Enabled Blockchain System for Home Care Service Innovation: Mixed Methods Study
Source: JMIR Med Inform. 2020 Jul 28;8(7):e15472. doi: 10.2196/15472 (PMC7420632; doi:10.2196/15472)
Supplement: Multimedia Appendix 3 [file medinform_v8i7e15472_app3.pdf]

Multimedia Appendix 3. Step-by-step workflow comparisons between existing and proposed systems.

Home care service workflow	Existing System	Blockchain-based system
1.Home care service demand (update to matching engine)	Human workforce for case filing	Demand updates by platform clients application
2.Case matching	Matching Engine	◎ Matching results on chain
2-1.Service notification to caregiver	Phone calls or Emails	◎ Ⓐ ⓒ
2-2.Service notification to caregiver	Phone calls or Emails	◎ Ⓐ
2-3.Decision making (caregiver)	Passive	Ⓜ Dominated by caregiver
2-4.Decision making (caretaker)	Passive	Ⓜ Dominated by caretaker
2-5.Service Confirmation	Care center intermediation	Ⓐ Smart-contract-enabled check/communication
3.Service Notification	Phone calls or Emails	◎ Ⓐ Facilitated via event-driven mechanism
4.Insurance application (policy)	Lack of short-term insurance products	Ⓐ ⓒ System generation accordin[illegible] to insurance conditions
5.Service assignment	Human workforce	Ⓐ ＊
6.Service delivery	Opaque monitor; Need further check	Ⓐ ＊
6-1 Way to caretaker’s	Poor control on process monitoring	＊
6-2 Service started		＊ Ⓐ
6-3 Back to caregiver’s		＊
6-4 End of service		＊ Ⓐ
6-5 Claims for accidents	Risks of counterfeit	ⓒ
7. Insurance cancellation	Human termination	Ⓐ Ⓜ
8. End of care service case	Human termination	Ⓐ

Note: Transparency ◎; Traceability ＊;. Level of automation Ⓐ; Counterfeit/Fraud-proof ⓒ; Management of insurance/welfare Ⓜ.
