# Supplementary material for: Development and Evaluation of a Smart Contract–Enabled Blockchain System for Home Care Service Innovation: Mixed Methods Study
Source: JMIR Med Inform. 2020 Jul 28;8(7):e15472. doi: 10.2196/15472 (PMC7420632; doi:10.2196/15472)
Supplement: Multimedia Appendix 4 [file medinform_v8i7e15472_app4.docx]

**Multimedia Appendix 4.** Home Care Service System Questionnaire.

Date: _______________

Part I. Introduction

**Dear Respondent,**

**The purpose of this questionnaire is to help understand your opinion on homecare service systems. Recent studies have reported issues in processing demand/supply information, service matching, employment, and insurance of homecare service. Meanwhile, potential influences may bring new opportunities and challenges when adopting new information technology, such as “blockchain.” Through this brief survey, your answers help enhance academic research. Your response will ONLY be used for academic purposes. Thank you for your time and opinions.**

Part II. Demographic Data

| A. Gender | | | | | | | | | | | | |
| --- | --- | --- | --- | --- | --- | --- | --- | --- | --- | --- | --- | --- |
| □ Male | | | | | | □ Female | | | | | | |
| B. Age | | | | | | | | | | | | |
| - <20 | □ 21–30 | | □ 31–40 | | □ 41–50 | | | □ 51–60 | | □ 61–70 | | □ >70 |
| C. Education | | | | | | | | | | | | |
| - Under Senior | | □ Vocational | | □ Bachelor | | | | | □ Master | | □ Doctorate | |
| D. Occupation | | | | | | |  | | | | | |
| □ Student | | □ Scholar | | □ Civil servant | | | | | □ Engineer | | □ Financial | |
| □ Health care | | □ Social worker | | □ Caregiver | | | | | □ Care center | | □ Others______ | |
| E. Level of understanding of FinTech (e.g., bitcoin, digital wallet, blockchain, and smart contract) | | | | | | | | | | | | |
| □ None or Poor | | □ Little | | □ Fair | | | | | □ Good | | □ Specialized | |

Part III. Questionnaire Items

The questionnaire is divided into five parts, namely, **Care center, Caregiver, Caretaker, Insurance Company, and Public Sector**. Four main indicators, namely, traceability, efficiency, level of automation, and easiness of management, comprise the categories of measured items. The following items (15 in total) describe statements about the critical function/capability of the homecare service system. Please indicate your response (level of agreement or disagreement) to the following statements using this scale.

| **1** | **2** | **3** | **4** | **5** |
| --- | --- | --- | --- | --- |
| Strongly Disagree | Disagree | Neutral | Agree | Strongly Agree |

| **A. Care Center** | | | | | | |
| --- | --- | --- | --- | --- | --- | --- |
| **Items and Statements** | **System** | **SD SA** | | | | |
| 1. The system will enable the tracking of care service assignment and service provided by the caregiver. | Existing | 1 | 2 | 3 | 4 | 5 |
|  | Blockchain | 1 | 2 | 3 | 4 | 5 |
| 2. The system allows effective monitoring and rapid retrieval of information on caretaker status and conducts corresponding feedback. | Existing | 1 | 2 | 3 | 4 | 5 |
|  | Blockchain | 1 | 2 | 3 | 4 | 5 |
| 3. The system is useful in filing insurance application/claims and makes related procedures efficient. | Existing | 1 | 2 | 3 | 4 | 5 |
|  | Blockchain | 1 | 2 | 3 | 4 | 5 |
| 4. The system enables process automation on short-term care insurance application and claims. | Existing | 1 | 2 | 3 | 4 | 5 |
|  | Blockchain | 1 | 2 | 3 | 4 | 5 |
| **B. Caregiver** | | | | | | |
| 5. The system allows traceability of caregiver matching results, employment, and service status. | Existing | 1 | 2 | 3 | 4 | 5 |
|  | Blockchain | 1 | 2 | 3 | 4 | 5 |
| 6. The system enables efficient notifications to the care center when accidents occur during care service. | Existing | 1 | 2 | 3 | 4 | 5 |
|  | Blockchain | 1 | 2 | 3 | 4 | 5 |
| 7. The system allows automatic update on service status, activates emergency notifications to the care center, and completes insurance claims procedures. | Existing | 1 | 2 | 3 | 4 | 5 |
|  | Blockchain | 1 | 2 | 3 | 4 | 5 |
| **C. Caretaker** | | | | | | |
| 8. The system makes necessary care service available for the caretaker. | Existing | 1 | 2 | 3 | 4 | 5 |
|  | Blockchain | 1 | 2 | 3 | 4 | 5 |
| 9. The system ensures that the caretaker receives efficient insurance claims when accidents occur. | Existing | 1 | 2 | 3 | 4 | 5 |
|  | Blockchain | 1 | 2 | 3 | 4 | 5 |
| **D. Insurance Company** | | | | | | |
| 10. The system enables the insurance company to track the current status of the insurance policy and if certain claim conditions occur. | Existing | 1 | 2 | 3 | 4 | 5 |
|  | Blockchain | 1 | 2 | 3 | 4 | 5 |
| 11. The system ensures better efficiency when the insurance company enforces related processes of policy application/cancellation and insurance claims. | Existing | 1 | 2 | 3 | 4 | 5 |
|  | Blockchain | 1 | 2 | 3 | 4 | 5 |
| 12. The system is able to conduct short-term policy application/cancellation and insurance claims automatically. | Existing | 1 | 2 | 3 | 4 | 5 |
|  | Blockchain | 1 | 2 | 3 | 4 | 5 |
| 13. The system makes the insurance company simplify sophisticated procedures in managing policy application/cancellation and insurance claims | Existing | 1 | 2 | 3 | 4 | 5 |
|  | Blockchain | 1 | 2 | 3 | 4 | 5 |
| **E. Public Sector** | | | | | | |
| 14. The system ensures that the public sector has better capability to track data on service matching, employment, and insurance. | Existing | 1 | 2 | 3 | 4 | 5 |
|  | Blockchain | 1 | 2 | 3 | 4 | 5 |
| 15. The system enables the public sector to monitor the matching and provision of homecare service. | Existing | 1 | 2 | 3 | 4 | 5 |
|  | Blockchain | 1 | 2 | 3 | 4 | 5 |

Part IV. Comments and Overall Evaluation

A. Your preferences on using existing or blockchain-based homecare system.

□ Existing System □ Blockchain-enabled Homecare System

B. Please give your opinion on the level of importance of the four main indicators (a. Traceability, b. Efficiency, c. Level of automation, d. Easiness of Management)
